# Carbohydrate-mediated responses during zygotic and early somatic embryogenesis in the endangered conifer, *Araucaria angustifolia*

**DOI:** 10.1371/journal.pone.0180051

**Published:** 2017-07-05

**Authors:** Bruno V. Navarro, Paula Elbl, Amanda P. De Souza, Vinicius Jardim, Leandro F. de Oliveira, Amanda F. Macedo, André L. W. dos Santos, Marcos S. Buckeridge, Eny I. S. Floh

**Affiliations:** 1 Laboratory of Plant Cell Biology, Department of Botany, Institute of Biosciences, University of São Paulo, São Paulo-SP, Brazil; 2 Laboratory of Plant Physiological Ecology, Department of Botany, Institute of Biosciences, University of São Paulo, São Paulo-SP, Brazil; McGill University, CANADA

## Abstract

Three zygotic developmental stages and two somatic *Araucaria angustifolia* cell lines with contrasting embryogenic potential were analyzed to identify the carbohydrate-mediated responses associated with embryo formation. Using a comparison between zygotic and somatic embryogenesis systems, the non-structural carbohydrate content, cell wall sugar composition and expression of genes involved in sugar sensing were analyzed, and a network analysis was used to identify coordinated features during embryogenesis. We observed that carbohydrate-mediated responses occur mainly during the early stages of zygotic embryo formation, and that during seed development there are coordinated changes that affect the development of the different structures (embryo and megagametophyte). Furthermore, sucrose and starch accumulation were associated with the responsiveness of the cell lines. This study sheds light on how carbohydrate metabolism is influenced during zygotic and somatic embryogenesis in the endangered conifer species, *A*. *angustifolia*.

## Introduction

Embryogenesis is a spatio-temporally organized developmental process that is central to, and conserved among, the life cycles of diverse plant species. It plays a key role in defining many aspects of seed development and diversity [1,2]. The zygote produces the first tissue precursors from a single totipotent cell, as well as the first stem cells, and by the end of embryogenesis the zygote has transformed into a fully mature embryo [3]. Embryogenesis therefore provides an excellent system in which to study the progression from the very first cell and tissue type specification events to multicellular tissue formation. However, much remains to be learnt about the regulatory mechanisms involved in plant embryo development, and particularly the early stages of embryogenesis [4].

*In vitro* somatic embryogenesis is recognized as not only a method for regenerating entire plants, but also as a potential system for analyzing the regulation of gene expression, and the metabolite and morphological changes that occur during embryo development [5,6]. Somatic embryogenesis is thought to be analogous to zygotic embryogenesis, and a number of morphological, physiological, biochemical and molecular similarities have been identified [5,7,8,9]. In addition, somatic embryogenesis represents a highly desirable *in vitro* propagation system, since it can yield a large number of plants and can be coupled with cryopreservation, bioreactors, synthetic seed technologies and genetic transformation [10], in order to provide increase production and to subsidize scientific researches.

In the case of conifers, somatic embryogenesis is a valuable supplement to conventional plant propagation and breeding approaches [11,12]. It provides a reliable experimental system for investigating the regulatory mechanisms of embryo development [13,14]. *Araucaria angustifolia*, a native Brazilian conifer with considerable economic, ecological and social importance [15], has emerged as a model for investigating embryo development. This species is classified as critically endangered by the International Union of Conservation of Nature Red List of Threatened Species [16] and has recalcitrant seeds, resulting in rapid loss of viability [17]. In order to enhance the *in vitro* propagation of *A*. *angustifolia* via somatic embryogenesis, previous studies have analyzed molecular and physiological processes associated with both *A*. *angustifolia* zygotic and somatic embryogenesis [17]. However, in contrast to many other conifers [13,18,19,20,21], a protocol for the efficient regeneration of *A*. *angustifolia* through somatic embryogenesis has not yet been developed. In part, this is due to insufficient knowledge of the underlying regulatory process that control embryogeneis in this species [15,22,23,24].

Various studies have collectively evaluated patterns of gene expression and/or the regulation of different metabolites during *A*. *angustifolia* embryogenesis [23,24,25,26]. A notable exception is the role of carbohydrates, which has not been studied in detail, although transcriptome and proteome analyses suggest that several genes and proteins involved in carbohydrate metabolism are highly expressed during both zygotic and somatic embryo development [15,22]. In addition, while the importance of carbohydrate metabolism and nutrient availability for somatic embryo growth and development has been examined in other conifer species [11,13,27,28,29], a role for carbohydrates as signaling molecules during embryogenesis has not been established.

Carbohydrates can act as signaling molecules and regulators of gene expression, as part of signaling networks connecting the environment with plant metabolism, development and growth [30,31]. Among the known carbohydrate-mediated signaling molecules, TOR (target of rapamycin), a Ser/Thr protein kinase that perceives nutrient availability and direct growth and metabolic patterns, has emerged as a central coordinator of nutrient and energy status [32,33,34], promoting growth and development in responses to high carbon availability. In contrast, Snf1-related kinase 1 (SnRK1) is active upon sugar deprivation [35]. Both TOR and SnRK1 activities are modulated by sugar status, thereby promoting the coordination of energy consumption and preservation. This, in turn, is linked to adaptations to stress conditions [36,37], which are known to be sensed by several signaling processes and molecules [35].

Trehalose-6-phosphate (T6P), an intermediary carbohydrate in the trehalose biosynthesis pathway, is essential for plant growth, and acts as a signal of sugar availability, linking growth and development to carbon status [38,36]. In a two-step pathway, trehalose-6-phosphate synthase (TPS) converts glucose-6-phosphate and UDP-glucose to the growth-inducing signaling molecule T6P. T6P catabolism by trehalose-6-phosphate phosphatase (TPP) yields trehalose, which is further hydrolyzed to glucose by trehalase [35]. The relationship between T6P and SnRK1 in developing tissues is complex and has not yet been fully elucidated, although it is thought to involve both direct and indirect mechanisms [38]. No direct connection between T6P and TOR has yet been described. However, both act in response to high sugar status [35], consistent with the idea of a regulatory network involving TOR, SnRK1 and T6P signaling. To date, plant sugar signaling molecules and associated sensors have been shown to play a role in embryogenesis, seedling establishment, growth, metabolism, juvenile-adult transition, flowering and senescence [39]. Nonetheless, most of these studies have focused on *Arabidopsis thaliana* and other model species with short life cycles, rather than perennial species, such as *A*. *angustifolia*.

Previous studies of *A*. *angustifolia* have not addressed possible relationships between gene expression and metabolic switches in the context of carbohydrate metabolism and sugar sensing. Here, we examined the expression patterns of putative key genes involved in carbohydrate-mediated growth regulation in *A*. *angustifolia* during zygotic and somatic embryos. Specifically, we measured the levels of non-structural carbohydrates and characterized cell wall composition, together with quantitative PCR (qRT-PCR) analyses of the expression of genes involved in sugar sensing and trehalose biosynthesis, in three different seed developmental stages and two somatic cell lines, in the proliferation and maturation phases. We present evidences, for the first time in a conifer species, that sugar sensing and signaling, known to be important for the formation of the zygotic embryo, are also involved in somatic embryo development. We also identify possible markers for embryo responsiveness and quality.

## Materials and methods

### Plant materials

**Fig 1:**
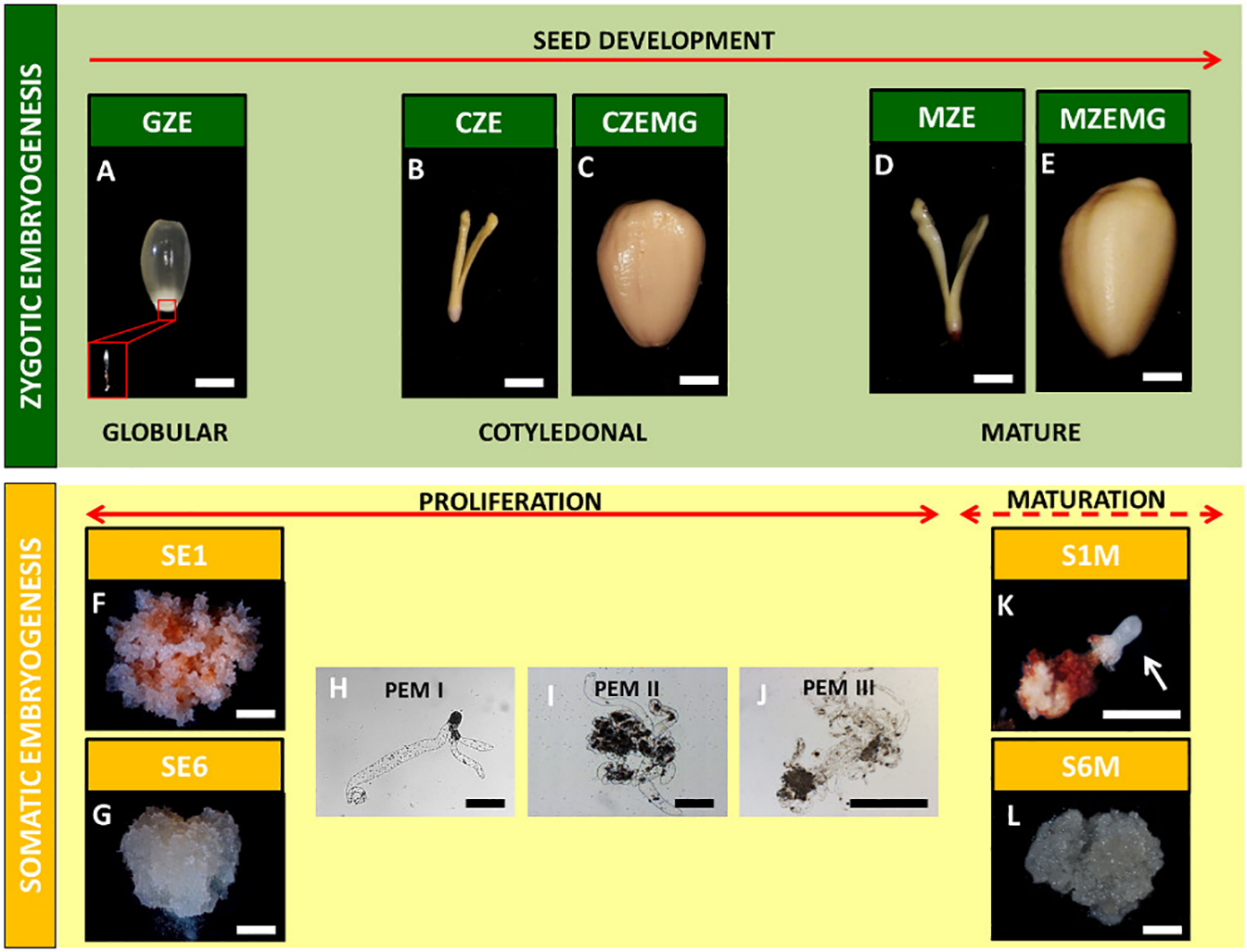
*Araucaria angustifolia* zygotic and somatic samples used in this study. Globular zygotic embryo with megagametophyte (GZE) (a); cotyledonal zygotic embryo (CZE) (b) and the corresponding megagametophyte (CZEMG) (c); mature zygotic embryo (MZE) (d) and the corresponding megagametophyte (MZEMG) (e); abscisic acid (ABA)-responsive (SE1) (f) and ABA-blocked (SE6) (g) cell lines in proliferation medium, and the development of proembryogenic masses (PEMs) (h, i and j); mature ABA-responsive (S1M) (k) and mature ABA-blocked (S6M) (l) embryogenic cell lines. Arrow indicates globular somatic embryo. Scale bars: a-g, l = 5 mm; h-j = 0.2 mm = 10 mm.

Three developmental stages of *A*. *angustifolia* zygotic embryos (Fig 1A–1E) and two embryogenic cultures in proliferation (Fig 1F and 1G) and maturation (Fig 1H and 1I) medium were harvested and sampled as previously described [40]. The zygotic embryogenesis samples were: a) the globular zygotic embryos (GZE); b) cotyledonal zygotic embryos (CZE); c) megagametophytes of the cotyledonal embryos (CZEMG); d) mature zygotic embryos (MZE); and e) megagametophytes of the mature embryo (MZEMG). Samples of GZE also include their correspondent megagametophyte since at this stage of development, the isolation of the embryo from megagametophyte is not possible given the small size of it.

The somatic embryogenesis samples consisted of two embryogenic cell lines (EC) previously described by Jo *et al*. [23] as ABA-responsive (SE1) and blocked (SE6). These two cell lines were analyzed under two *in vitro* conditions: a) during the proliferation phase, and b) during the maturation phase (S1M and S6M). The sample S1M was composed by the early somatic embryos in the globular stage, as well as its respective non-responsive tissue.

All samples were separated into three biological replicates for analyses of biochemical composition and gene expression.

### Database searches and phylogenetic analyses

The *A*. *angustifolia* transcriptome database [22] was surveyed using tBLASTn (E-value > e^-10^) [41] searches with the corresponding *A*. *thaliana* protein sequences of TOR (target of rapamycin), RAPTOR (regulatory associate protein of TOR), LST8 (lethal with sec-13 protein8), SnRK1 (plant Snfl1-related kinase 1), UGP (UDP-glucose pyrophosphorylase), TPS (trehalose-6-phosphate synthase) and TPP (trehalose-6-phosphate phosphatase). *A*. *thaliana* gene ID used in this work are detailed in S1 Table. Phylogenetic analyses were performed using other plant homologs (S1 Table) of the protein sequences described above, obtained by searching the Phytozome [42], Uniprot [43], Gymno PLAZA 1.0 [44] and SustainPineDB [45] databases (S1 Table). The sequences were aligned using the MUSCLE/CLUSTALW program with default parameters [46]. The alignment was analyzed using the Neighbor-Joining method [47], the distances were calculated using the JTT model and the tree topology was drawn by Subtree Pruning and Regrafting (SPR) with branch support values improved by the approximate likelihood ratio test (aLRT) [48]. Data associated with this analysis are shown in S1 Appendix.

### qRT-PCR analysis

RNA extraction, DNAse treatment, cDNA synthesis, primer design and quantitative RT-PCR (qRT-PCR) analysis was performed as in Elbl *et al*. [40]. Gene-specific primers (S2 Table) used in the qRT-PCR assay were designed using the Oligo Perfect 3.1 program [49] according to MIQE guidelines [50]. The Cq values from two technical replicates and the primer efficiency were calculated using the LinRegPCR software [51]. The expression values of the target genes were normalized against the geometric average of the *AaEIF4B-L* (translational initiation factor 4B) and *AaPP2A* (protein phosphatase 2A) reference genes [40]. The relative expression of all the genes tested was calculated based on the average expression levels in the GZE sample and presented as Log_2_ fold changes.

### Non-structural carbohydrates and starch measurements

The total non-structural carbohydrates (NSC) and starch content was quantified as described by De Souza *et al*. [52]. For NSC extraction, samples were lyophilized and ground to a fine powder. Forty grams of each sample were subjected to ten consecutive washes with 1 mL of 80% ethanol (v/v) at 80°C. After each extraction, the samples were centrifuged (10,000 × *g*, 5 min), and the supernatants were dried under vacuum and re-suspended in deionized water. Aliquots of each sample were analyzed by high-performance anion exchange chromatography with pulsed amperometric detection (HPAEC/PAD) on a Carbopac PA1 column (Dionex ICS3000, Dionex, CA, USA). The separation of glucose, fructose, sucrose, myo-inositol and raffinose was achieved with an isocratic (100 mM NaOH) elution of sugars.

For starch extraction and quantification, the pellets obtained after ethanol extraction were washed with distilled water and dried for 4 h at 60°C. The dried material was treated with α-amylase (120 U mL^-1^) from *Bacillus licheniformis* and amyloglucosidase from *Aspergillus niger* (both from Megazyme^®^, Bray, Co. Wicklow, Ireland). This procedure was repeated once and the reaction was stopped by addition of 50 μL of 0.8 M perchloric acid. Aliquots of the supernatant were incubated with 250 μL glucose oxidase/peroxidase (GOD/POD) (Labtest®, MG, Brazil) at 30°C for 15 min, and the glucose content released during enzymatic reactions was determined using a microplate reader at 490 nm. A standard curve was prepared using glucose solutions ranging from 20 to 300 mg mL^-1^. Glucose content was converted into starch content considering the starch being 90% of the total measured glucose [53]. Results were expressed in mg g^-1^ of dry weight (DW).

### Cell wall sugars

After the NSC and starch extraction, the alcohol insoluble residue (AIR) pellets were used for cell wall sugar analyses. Two milligrams of AIR were subjected to acid hydrolysis in 1 mL of 2M trifluoroacetic acid (TFA) for 1h at 100°C in a dry bath. The supernatants were collected and dried under vacuum before being re-suspended in 500 μL of deionized water. The monosaccharides arabinose, fucose, galactose, glucose, mannose, rhamnose and xylose were analyzed using a HPAEC/PAD system on a Carbopac SA10 column (Dionex-DX500, Dionex, CA, USA) as described by De Souza *et al*. [54] and the values expressed as a percentage (%) of total sugar.

### Statistical and correlation analysis

Data were analyzed by analysis of variance (ANOVA) followed by a Tukey’s test (*p*<0.01). When appropriate, data were transformed using the log2 function. The analyses were carried out using R version 3.2.2 [55]. PCA analysis was performed using the FactoMiner R package [56]. For network analysis, Pearson’s correlations were calculated in the R Stats package [57] and networks were built and drawn using the R Igraph package [58]. The links between the nodes (i.e. genes and carbohydrates) were created only when the correlation coefficient was > 0.9.

## Results

### Zygotic and somatic embryo development are morphologically similar

Three different stages of *A*. *angustifolia* seed development and two *A*. *angustifolia* cell lines with different embryogenic capacities were used in this study, to compare the embryogenic events related to carbohydrate metabolism in both zygotic and somatic embryogenesis (Fig 1). During zygotic embryogenesis, the globular stage (GZE) exhibited an immature embryo with a suspensor attached to the embryonic axis, and a translucent and mucilaginous megagametophyte (Fig 1A). Due to the small embryo size at this stage, the embryo and the megagametophyte were analyzed together. Subsequently, in the cotyledonal stage, the embryo (CZE) had developed cotyledon structures and a megagametophyte (CZEMG) with storage reserve deposits (Fig 1B and 1C). In the transition from the cotyledonal stage to the mature stage, no morphological changes were observed, and the only notable events were growth and elongation of the mature zygotic embryo (MZE) and the mature megagametophyte (MZEMG) (Fig 1D and 1E).

The somatic cell lines were characterized by their different embryogenic capacities, according to Jo *et al*. [23]: responsive to (SE1) and blocked (SE6) by exogenous abscisic acid (ABA) added to the medium during the maturation phase. Both cell lines showed translucent and mucilaginous cell masses during the proliferation phase (Fig 1F and 1G) and were formed by proembryogenic masses (PEMs), which consisted of meristematic cells with a dense cytoplasm and vacuolated and highly elongated suspensor cells (Fig 1H, 1I and 1J). Both cell lines were transferred to maturation medium (S1M and S6M), in the presence of osmotic agents and ABA. However, only the responsive cell line (S1M) was able to form early somatic embryos in the maturation medium. These early somatic embryos were characterized by spherical opaque embryonic heads and a degraded suspensor cell region (Fig 1K). The blocked cell line did not show the development of early somatic embryos (Fig 1L). These responsive and blocked cell lines were used as contrasting systems to compare with *A*. *angustifolia* zygotic embryo development in the subsequent analyses.

### Zygotic and somatic embryogenesis show different patterns of non-structural carbohydrate accumulation

We examined the spatial and temporal variation in carbohydrate composition in the three developmental stages of the zygotic embryos (GZE, CZE and MZE), their respective megagametophytes (CZEMG and MZEMG), and the two embryogenic cultures grown in proliferation (SE1 and SE6) and maturation (S1M and S6M) media (Fig 1) [22,23]. Specifically, we quantified the levels of NSC and evaluated cell wall composition.

**Fig 2:**
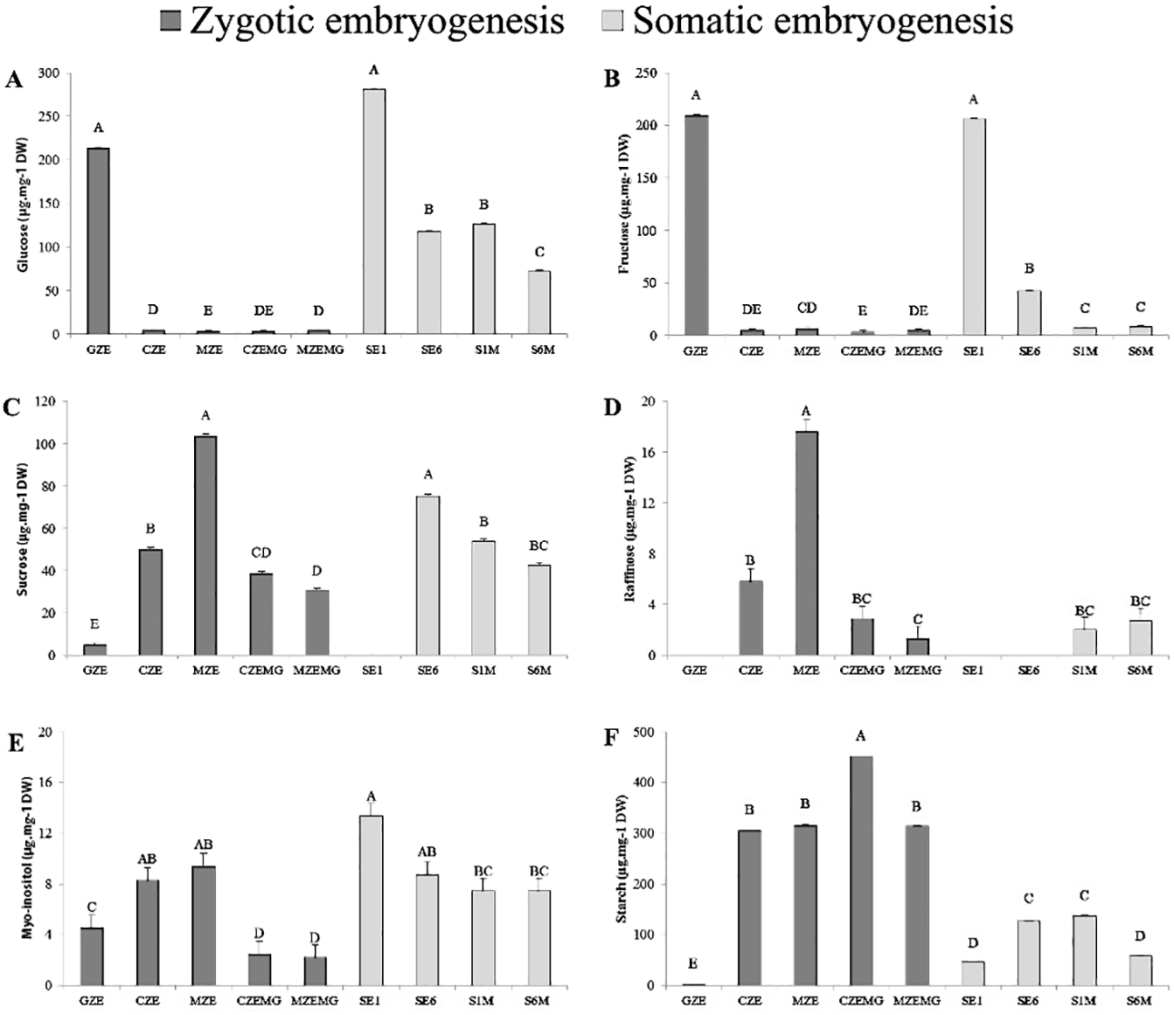
Glucose (a), fructose (b), sucrose (c), raffinose (d), *myo*-inositol (e) and starch (f) content (μg.mg-1 dry weight) of *A*. *angustifolia* globular (GZE), cotyledonal (CZE) and mature (MZE) zygotic embryos; cotyledonal (CZEMG) and mature (MZEMG) zygotic megagametophytes; and two embryogenic cultures in the proliferation (SE1 and SE6) and maturation (S1M and S6M) phase. Values are presented as averages ± standard error. Means followed by uppercase letters are significantly different among the samples, according to the Tukey’s test (P<0.05).

GZE and SE1 were characterized by high levels of hexoses (glucose and fructose), which decreased by 98% during seed development, and by 73% and 50% when the SE1 and SE6 cell lines, respectively, were transferred to maturation medium (Fig 2A and 2B). Sucrose levels showed the opposite trend, increasing throughout zygotic embryo development (Fig 2C). The SE1 cell line exhibited a similar pattern to zygotic embryo, with sucrose predominating over the hexoses in the maturation phase; S6M, however, had 43% less sucrose than the S1M in proliferation medium.

Similar to sucrose, raffinose content increased during zygotic embryogenesis. During GZE and the proliferation phase of the somatic cell lines, raffinose was not detected (Fig 2D), but in subsequent stages and in the maturation phase, raffinose levels ranged from 1.3–17.6 mg g^-1^ of dry weight (DW). The amount of *myo*-inositol increased by 52% during zygotic embryo development, while it decreased 45% from responsive cell line grown in proliferation medium (SE1) to responsive cell line grown in maturation medium (S1M) (Fig 2E, S3 Table).

Starch was the main NSC and was particularly prevalent in the megagametophytes (CZEMG and MZEMG), representing almost 50% of the seed dry weight in the cotyledonal stage (Fig 2F). Starch accumulation started in the GZE and increased during seed development. Starch degradation was apparent in megagametophytes (CZEMG and MZEMG) during late embryogenesis, and in the blocked cell line during the transition from proliferation (SE6) to maturation (S6M) medium.

During development, we observed no changes in the relative proportions of cell wall sugars in either the zygotic and somatic embryos (S1 Fig). Arabinose and galactose were the most abundant, accounting for 73% of the total quantified monosaccharides.

The similarity of NSC profiles between zygotic embryos and the responsive cell line (SE1) suggests that a decline in hexose content, with a concomitant accumulation of sucrose and starch throughout development, are important for embryo formation. This idea is supported by the fact that in the blocked lines (SE6), the reduction in levels of hexoses from cells grown in proliferation medium compared to maturation medium was smaller, while sucrose and starch contents were lower.

### The expression patterns of sugar sensing- and trehalose biosynthesis- related genes change depending on the sugar status

**Fig 3:**
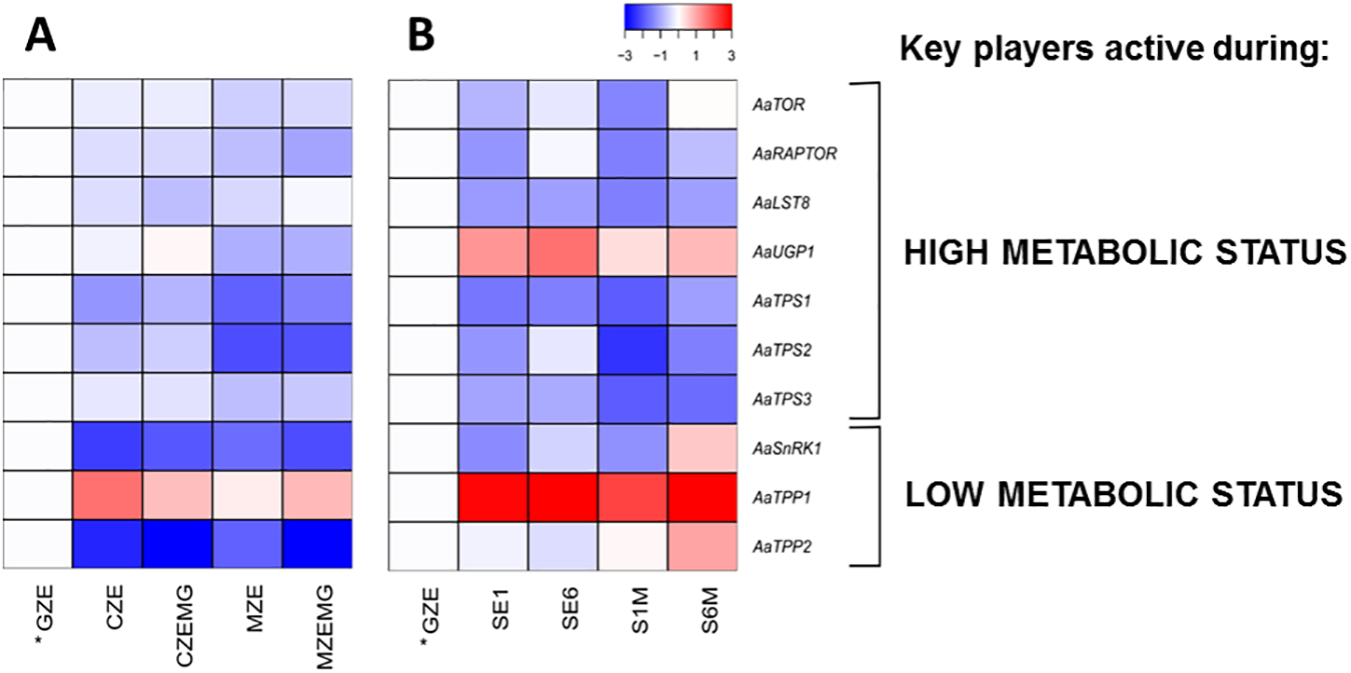
Heat maps of sugar sensing and trehalose biosynthesis pathway associated genes during *A*. *angustifolia* zygotic (a) and somatic (B) embryogenesis. The genes were divided into groups that are expressed during high (*AaTOR*, *AaRAPTOR*, *AaLST8*, *AaUGP1*, *AaTPS1*, *AaTPS2 and AaTPS3*) and low (*AaSnRK1*, *AaTPP1 and AaTPP2*) metabolic status. Expression levels in all the samples analyzed were normalized to those in the GZE sample. The heat maps showing expression patterns and gene expression values are shown in S5 Table.

We measured the expression of ten genes associated with sugar sensing and trehalose biosynthesis during *A*. *angustifolia* zygotic and somatic embryogenesis using qRT-PCR (S1 Appendix). The identity of all genes was confirmed by phylogenetic (S2 Fig) and alignment analyses (S3 Fig) (S1 Appendix), revealing high similarities with *A*. *thaliana* amino acids sequences (S4 Table). All values were normalized to those at the GZE stage. Fig 3 shows two heat maps of expression patterns related to zygotic and somatic embryogenesis, classified into genes expressed during high (*AaTOR*, *AaRAPTOR*, *AaLST8*, *AaUGP1*, *AaTPS1*, *AaTPS2* and *AaTPS3*) and low (*AaSnRK1*, *AaTPP1* and *AaTPP2*) metabolic sugar status [35]. Nine of the ten genes analyzed throughout the zygotic embryogenesis stages showed a decrease in their expression levels compared to the GZE stage (Fig 3A, S5 Table). However, during somatic embryogenesis, only six genes showed this pattern (Fig 3B). While the responsive cell line had similar gene expression patterns in the proliferation and maturation phase (SE1 and S1M, respectively), the blocked cell line showed more variation between these two phases (SE6 and S6M, respectively).

The genes that were expressed at higher levels during stages of high metabolic sugar status showed similar trends during zygotic (Fig 3A) and somatic (Fig 3B) embryogenesis, with the exception of *AaUGP1*, which was expressed at higher levels during somatic embryogenesis. However, for the genes that were active during low metabolic sugar status, the differences in expression between the zygotic and somatic samples were more evident. This was particularly notable for *AaSnRK1* and *AaTPP2*, which expression increased in the blocked cell line (SE6 and S6M) during the transition from proliferation to maturation medium (Fig 3B). We noted that *AaTPP1* and *AaTPP2* showed the opposite pattern expression during both zygotic and somatic embryogenesis. Taken together, the gene expression data suggest a difference in sugar sensing between *A*. *angustifolia* zygotic and somatic embryogenesis, which further suggests a possible association of the sugar sensing process with recalcitrance and sink/source tissue trade-off during embryo development.

### Changes in transcript levels of genes involved in sugar sensing and NSC contents during zygotic and somatic embryogenesis

**Fig 4:**
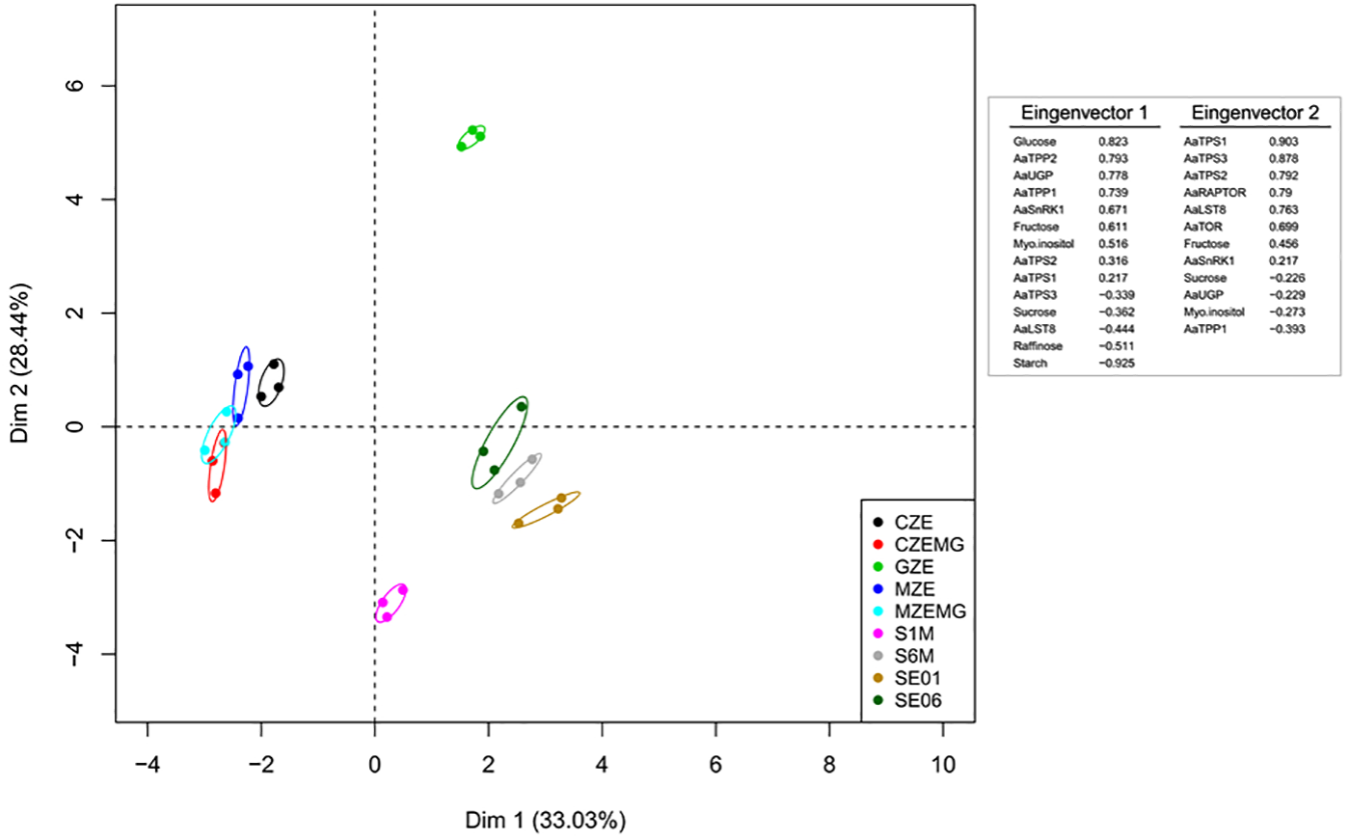
Principal component analysis (PCA) of transcripts and metabolic profiles of non-structural carbohydrates (NSC) and sugar sensing and trehalose biosynthetic pathway associated genes in three different *A*. *angustifolia* seed developmental stages and two embryogenic cell lines. GZE, megagametophytes containing globular embryos; CZE, isolated cotyledonal embryos; MZE, isolated mature embryos; CZEMG, megagametophytes at the cotyledonal stage; MZEMG, megagametophytes at the mature stage; SE1, ABA-responsive cell line; SE6, ABA-blocked cell line; S1M, ABA-responsive cell line in maturation phase; SE6, ABA-blocked cell line in maturation phase.

To better understand the trade-off between the variations in the mRNA levels of genes involved in sugar sensing and NSC contents, we used two different approaches. First, a principal component analysis (PCA) was performed (Fig 4). The first dimension (Dim1), which explains 33% of the data variance, separated the zygotic embryo development stages (CZE, CZEMG, MZE, and MZEMG) from the GZE stage and somatic cell lines (SE1, S1M, SE6, and S6M), while the second dimension (Dim2, 28%) showed that the GZE stage was distinct from the other developmental stages and the somatic cell lines.

**Fig 5:**
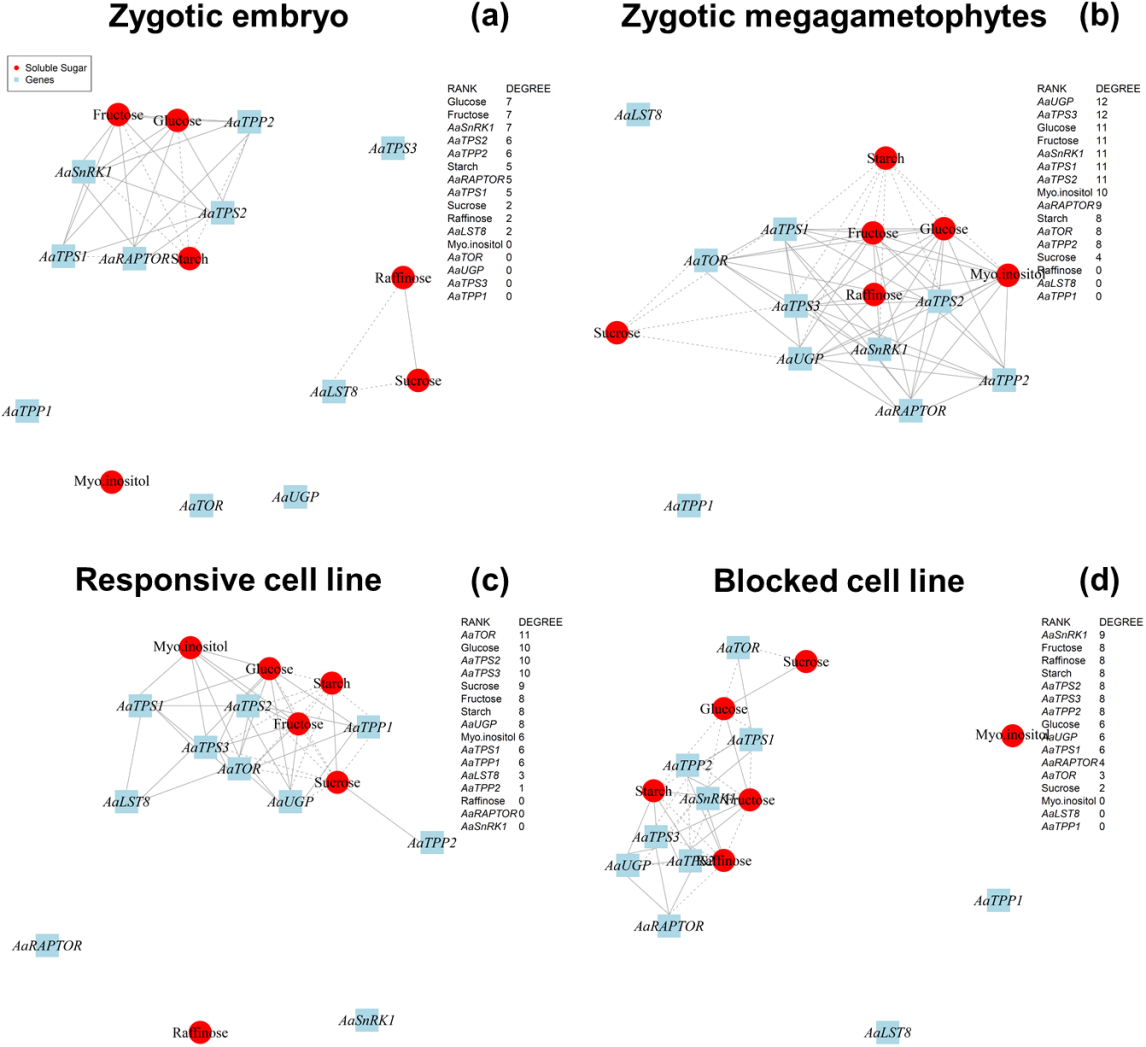
Co-variation network based on gene expression and non-structural carbohydrate (NSC) content. Red circles and blue boxes represent gene and metabolite nodes, respectively. Continuous and dashed lines represent positive and negative correlations, respectively. The network was constructed using the R Stats package [54] and networks were built and drawn using the R Igraph package [57].

The second approach involved a correlation network analysis (Fig 5). Four networks were built focusing on the development of different samples: 1) zygotic embryos (GZE, CZE and MZE) (Fig 5A); 2) zygotic megagametophytes (GZE, CZEMG and MZEMG) (Fig 5B); 3) the responsive cell line (SE1 and S1M) (Fig 5C); 4) and the blocked cell line (SE6 and S6M) (Fig 5D). We used degree centrality analysis to compare the networks, which assesses the number of links that each node (a substance or a gene) receives, and therefore reflects the potential importance of a given node in a particular network. The link represents a covariance between, or among different nodes. It also shows if the connections are positive or negative correlations.

In the zygotic embryo network, two isolated networks were observed, with glucose, fructose and *AaSnRK1* showing the highest number of links, as observed by high values of degree centrality (Fig 5A). In contrast, the zygotic megagametophytes showed a single network, involving *AaUGP* and *AaTPS3*, as intermediates in the trehalose biosynthesis pathway. Within these networks, levels of sucrose, glucose and fructose always had negative correlations with those of starch (Fig 5B). Unlike the zygotic networks, the network for the responsive cell line suggested that *AaTOR* is the main regulator as it had the highest degree of centrality (Fig 5C). Interestingly, *AaSnRK1*, which showed the opposite expression patterns to *AaTOR* in the qRT-PCR analysis, appeared to be central in the blocked cell line network (Fig 5D). These results suggest that these genes may play key roles in NSC carbon mobilization during *A*. *angustifolia* embryo development.

## Discussion

### Sugar sensing-mediated responses occur mainly during the early stages of zygotic embryogenesis

In conifers, the levels of storage compounds have a significant effect on embryo development [59,60], and carbohydrate storage reserves can accumulate in large amounts [27]. In this study, we investigated the changes in carbohydrate metabolism and transcript levels during *A*. *angustifolia* stage-specific zygotic embryo and megagametophyte tissue development.

The globular stage (GZE) was considered to be the starting point of *A*. *angustifolia* embryo development and at this stage, the seeds had high levels of hexoses and low levels of sucrose. This pattern changed during development and, in the late embryogenesis stages (cotyledonal and mature), a decrease in hexose content with an associated transient accumulation of sucrose was observed (Fig 2A, 2B and 2C). This increase in the sucrose:hexose ratio during embryo development appears to be a common trend in conifers as it was also observed in *Pinus taeda* [27] and *Picea abies* [61]. Sucrose accumulation is also thought to be a key factor in the carbohydrate metabolic status signaling pathway, and can control storage and differentiation processes through the regulation of metabolic enzymes, gene expression and enzyme activity [62].

Concomitant to increases in sucrose, raffinose accumulated during the later stages of seed maturation (Fig 1D). Raffinose family oligosaccharides (RFOs) play a protective role under stress conditions, acting as membrane stabilizers [29,63], free radical scavengers [64] and osmoprotectants in the desiccation tolerance of orthodox seeds [29,64,65]. In the case of *A*. *angustifolia*, a recalcitrant conifer species, this sugar might act as a cryoprotectant and osmoprotectant, since its accumulation occurs later during seed maturation. This phase coincides with the transition of seasons, from autumn to winter in temperate zones, when the temperature range varies between 15 and 20°C throughout the day. In *A*. *angustifolia*, ABA levels peak at early stages of embryo development, and decrease at the mature stage [24], suggesting that it may regulate the accumulation of raffinose. Indeed, it is well documented that ABA promotes this accumulation by increasing the level of galactinol synthase activity, an important enzyme involved in raffinose biosynthesis [66].

The growth and development of heterotrophic tissues, such as developing seeds, depend on supplies of photoassimilates from the leaves, or remobilization of starch and other storage reserves [38]. Two evolutionarily conserved protein kinases, SnRK1 and TOR, play central and antagonistic roles in growth regulation, connecting external signals to biological responses, such as transcription, translation, ribosome biogenesis, translocation of regulatory proteins, autophagy, and storage of reserve compounds [32,67,68,69,70]. In yeast and animals cells, TOR acts as the catalytic component in two high-molecular-mass complexes, named TORC1 and TORC2 [32,71]. Three major components of TORC1, which are TOR, small lethal with SEC13 protein 8 (LST8) and regulatory associated protein of TOR (RAPTOR), are present in land plants and algae [72,73,74]. In *A*. *angustifolia*, the expression of TORC gene components (*AaTOR*, *AaRAPTOR* and *AaLST8*) was higher at the globular stage, and decreased during seed development (Fig 3A, S5 Table), suggesting a role in early embryogenesis. In conifers, this phase corresponds to all stages after elongation of the suspensor and before establishment of the root meristem, with the arrival of the dominant embryo in the cavity of the corrosion, the elongation of the secondary suspensor system, and programmed cell death of the subordinate embryos [75]. A similar role during embryogenesis was also observed in *A*. *thaliana*, where the null *tor* mutant exhibits growth arrest at the 16- to 32-cell embryo stages and its TOR kinase domain alone can partially rescue early embryo lethality at the initial development stage [33]. Additionally, the *raptor1* mutant is arrested in embryo development, confirming the importance of the interaction among the complex components [76].

During times of high metabolic sugar status, TOR promotes growth, while SnRK1 is activated during low sugar conditions [32]. We observed that during *A*. *angustifolia* zygotic embryogenesis, *AaTOR* expression was higher than *AaSnRK1* expression at all stages (Fig 3A, S5 Table), indicating the maintenance of a high metabolic status during seed development. Indeed, the repression of SnRK1 in pea (*Pisum sativum)* embryos was reported to result in phenotypes that were insensitive to ABA signaling, affecting seed maturation and storage activity [77]. This interaction has not yet been elucidated in early embryogenesis.

Recent studies showed that SnRK1 may be regulated by T6P [78,79]. TP6 levels are, in turn, regulated by trehalose-6-phosphate synthase (TPS) and trehalose-6-phosphate phosphatase (TPP). We observed a decrease in the expression of *AaTPS1*, *AaTPS2* and *AaTPS3*, and an increase in the expression of *AaTPP1* during *A*. *angustifolia* seed development, suggesting regulation of T6P levels by TPS and TPP. Such a regulatory system might be influenced by the metabolic status of the embryos at different developmental stages. Indeed, similar features were observed in the *A*. *thaliana tps1* mutant, in which embryos are hindered at the torpedo stage [80], demonstrating the importance of trehalose metabolism, and consequently, of T6P [38,81].

Concerning the difference between *AaTPP1* and *AaTPP2* during the zygotic embryo development, Vandesteene *et al*. [82] showed that TPPs have cell- and tissue-specific expression in Viridiplantae. Due to the ancestral duplication that occurs in the Embryophyta group (S2G Fig), these genes showed subfunctioning along the evolution. For *A*. *angustifolia* it is plausible that the difference in the expression between *AaTPP1* and *AaTPP2* is a result of the particularity of the embryogenic tissues.

Our findings contribute to the understanding of sugar sensing in perennial species features that are distinct from those of annual species, such as recalcitrant seeds. For *A*. *angustifolia*, the results suggest that the control points related to carbohydrate status are mainly in the early stages of zygotic embryo development. We conclude that somatic embryogenesis can provide an experimental system for elucidating the early stages of embryogenesis, which cannot be studied *in vivo*, in addition to its use in germplasm conservation and genetic improvement [17,22,83].

### Signaling involved in sucrose and starch accumulation is essential for somatic embryogenetic development

We previously hypothesized from gene expression studies [15,22] that carbohydrate and nitrogen metabolism are important during the proliferation and maturation phases of *A*. *angustifolia* somatic embryogenesis, reflecting the storage sink characteristics of the mature embryo. Here, we investigated the role of both NSCs and the structural carbohydrates of the cell wall during *A*. *angustifolia* embryogenesis, comparing a cell line that was responsive to ABA, a blocked cell line, and the globular stage of zygotic embryogenesis, since the two cell lines were induced from immature zygotic embryos [84].

We observed no changes in cell wall monosaccharide composition between the cell lines or different developmental stages (S1 Fig). However, we noted that arabinose and galactose were the most abundant monosaccharides in all the samples analyzed. These two monosaccharides may have been derived from cell wall localized arabinogalactan proteins, since these proteoglycans have been identified in *A*. *angustifolia* somatic cell lines [84] and have been implicated in many processes involved in plant growth and development, including somatic embryogenesis [85].

Sucrose is the most effective carbohydrate, when added exogenously, at supporting the proliferation and maturation of conifer somatic embryos [29,86]. Endogenous carbohydrate status varies throughout the somatic embryogenesis of conifers [29], and can be used to identify cell lines with high-quality embryos [11,13,86]. During the transition from the proliferation to the maturation phase, *A*. *angustifolia* embryonal masses exhibited high hexose contents and almost no sucrose, resulting in a low sucrose:hexose ratio. The same was observed for the globular zygotic stage (Fig 2A, 2B and 2C and S3 Table). Similar results were reported for other conifer species, such as *P*. *pinaster* [87] and *P*. *abies* [29]. While the sucrose:hexose ratio was not significantly different for the two cell lines, the content of sucrose increased in the responsive line, but decreased in the blocked line (Fig 2C and S3 Table). In addition, the blocked line showed a decrease in starch levels during the maturation phase. Unlike the responsive line and zygotic embryogenesis, where starch accumulation occurred in the later stages of development, starch accumulation in the blocked line occurred in the proliferation phase (Fig 2F). To maintain the metabolic activity, this reserve was consumed in stress conditions, when ABA and osmotic agents were present during maturation. Starch accumulation in other conifer species occurs mainly one month into the maturation process [88] and maturing embryos usually undergo a transition from a metabolic sink (with a prevalence of hexoses) to storage, with a high sucrose:hexose ratio and starch accumulation [29,89]. The early accumulation of starch followed by its degradation during the maturation phase in the blocked *A*. *angustifolia* line may explain why this line does not develop.

The nature of the carbohydrate supply can reflect the signaling networks that control development [90,91], including somatic embryogenesis. Sugar sensing and signaling processes might respond to differences in the metabolic status, influencing growth rate and therefore the timing of assimilation and storage of nutrients [32]. The expression levels of key genes related to sugar sensing were similar during the proliferation and maturation phases of the responsive cell line (Fig 3B and S5 Table), suggesting that the maturation agents introduced into the culture medium do not interfere with their expression to stop the formation of the somatic embryos in the early stage (Fig 1H). The blocked cell line reached the storage during proliferation, making consumption of reserves, such as sucrose and starch, necessary during the maturation phase. The expression patterns of sugar sensing genes suggested a similarity between this cell line and the cotyledonal megagametophyte (Fig 3), and we concluded that the cell line mimics the reserve feature observed in the cotyledonal tissue, which maintain a constant metabolic activity after seed maturation. This is the first evidence of a sugar sensing process associated with somatic embryogenesis, and suggests that the embryogenic capacity can be associated with the carbohydrate assimilation potential.

The induction of *A*. *augustifolia* embryo formation *in vitro* is rather poor (<10% of the cell lines become responsive), and because its seeds are recalcitrant, it is important to find conservation strategies for this species. The results presented in this study suggest that the sugar sensing system is important in the induction of embryo formation, and this information may be used in future applications to preserve *A*. *angustifolia*.

### Zygotic and somatic embryogenesis share metabolic and transcriptional profiles

We analyzed metabolic and transcriptional changes during zygotic and somatic embryogenesis related to the sugar sensing process, and the results suggested that these metabolites and genes contribute to embryo development. Many of these changes indicate that the regulatory networks involved in growth and development are highly inter-connected at the gene expression and metabolite levels.

The expression of genes, such as *TORC*, *SnRK1*, *TPS* and *TPP*, during both zygotic and somatic embryogenesis indicated tissue-specificity. We conclude that during zygotic embryo development, hexose levels and *AaSnRK1* expression play a central role in the modulation of carbohydrate metabolism, while *AaUGP* is important in megagametophyte tissues. During the somatic embryogenesis process, the increase in the *AaUGP* expression could be related to mechanisms that demand energy, such as growth and differentiation, which occur with more intensity in *in vitro* conditions. *AaTOR* and *AaSnRK1* were located centrally in the ABA responsive and blocked somatic embryogenesis cell lines, respectively, suggesting that embryogenic capacity is correlated with sugar sensing.

**Fig 6:**
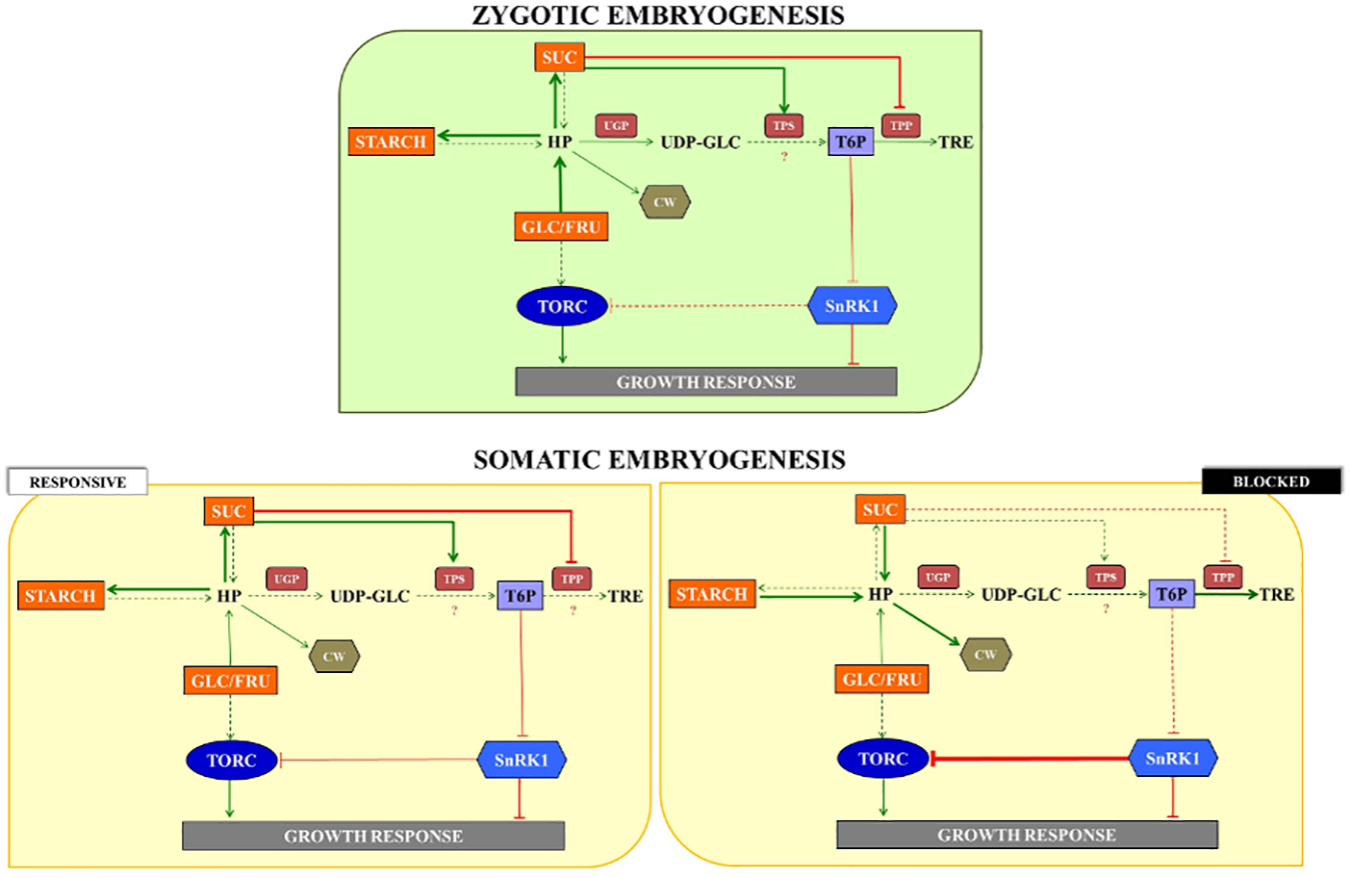
A simplified model of sugar sensing key players regulation by carbohydrates contents, acting growth responses, during the zygotic embryo development, as well as for the contrasting embryogenic cell lines. Continuous arrows represent the flux of the compounds, as well as the dashed arrows represent the opposite. The intensity of the flux is illustrated by the thickness of the arrows. Question marks indicate points in the model that are not explained by the data. Elements written in black represent substrates that were not measured in this work. HP: hexose phosphate; UDP-GLC: UDP-glucose; T6P: trehalose-6-phosphate; TRE: trehalose; CW: cell wall; TORC: TOR complex; SUC: sucrose; GLC/FRU: glucose/fructose.

Even though the complete mechanism of *A*. *angustifolia* somatic embryogenesis has not been determined in our study, there is another highly relevant implication of the carbon partitioning observations from this study. As illustrated in Fig 5, sugar sensing is a central process in the regulation of carbohydrates-mediated responses during *A*. *angustifolia* embryogenesis. Based on NSC contents and the gene expression, a simplified model was built, highlighting the traits concerning zygotic and somatic embryogenesis. These finds showed a possible trait-off centered between reserve accumulation (mainly, sucrose and starch) and *AaSnRK* expression (Fig 6). During the zygotic embryo development, the sucrose and starch levels increased exponentially (Fig 2C and 2F), while the *AaSnRK* expression decreased (Fig 3). T6P is essential for plant growth [92], acting in the inhibition of SnRK1 activity [32]. In *A*. *thaliana*, sucrose and T6P levels are positively correlated [35, 38], an evidence that in *A*. *angustifolia* the levels of T6P are responsible to inhibit the SnRK1 activity. A similar pattern was observed in the responsive cell line. However, the reduction in *AaSnRK* expression level between the proliferation and maturation phase in the responsive line was subtle, suggesting that the decrease in *AaSnRK* expression may be related to the quality of the somatic embryos. For the blocked cell line an inversed pattern with a decrease in sucrose and starch and an increase in *AaSnRK* expression was observed.

In conclusion, the trends in gene expression related to sugar sensing-mediated responses, identified during zygotic embryo formation were similar to those in the responsive cell line. Thus, we expect that the use of sugar sensing genes as molecular markers have potential for use in responsive cell line selection. Furthermore, the manipulation of factors that improve the somatic embryo development (i.e. culture medium and phytoregulators) may allow the modulation of sugar sensing responses for improved embryo development.

## Supporting information

S1 Fig Cell wall monosaccharides composition (%) of zygotic embryo stages–GZE (a), CZE (b), MZE (c), CZEMG (d) and MZEMG (e)–and two embryogenic cultures in proliferation–SE1 (f) and SE6 (g)–and maturation–S1M (h) and S6M (i)–phase of *A*. *angustifolia*.
(DOCX)

S2 Fig Phylogenetic trees constructed from sequences with homology to *Araucaria angustifolia* TOR (a), RAPTOR (b), LST8 (c), SnRK1 (d), UGP (e), TPS (f) and TPP (g). For TPS and TPP, the phylogenetic trees were constructed based on previous studies of [91] and [81], respectively. The trees were built with the maximum likelihood method using PhyML program [48] based on a multiple sequence alignment generated by MEGA 6.0 [46]. The evolutionary mode was estimated applying JTT substitution model and the tree topology was performed by Subtree Pruning and Regrafting (SPR) and the branch support values was improved by approximate likelihood ratio test (aLRT). The colors green, light brown and red represents the Viridiplantae, Fungi and Animalia clades, respectively. Database and accession numbers are listed in S1 Table.
(DOCX)

S3 Fig TOR (a), RAPTOR (b), LST8 (c), SnRK1 (d), UGP (e), TPS (f) and TPP (g) domains multiple proteins sequences alignments. Shading indicates homology (black 90–100%, grey 70–90%) and species with accession numbers are available at S1 Table.
(DOCX)

S1 Table Sequences used for construction of phylogenetic trees. (DOCX)

S2 Table List of primer sequences used in the qRT-PCR analysis of the sugar sensing associated genes. (DOCX)

S3 Table Non-Structural carbohydrates (NSC) content (μg.mg-1 dry weight) of zygotic embryo stages (GZE, CZE, MZE, CZEMG and MZEMG) and two embryogenic cultures in proliferation (SE1 and SE6) and maturation (S1M and S6M) phase of *A. angustifolia*. Values are presented in average ± standard error.
(DOCX)

S4 Table Comparison of *Araucaria angustifolia* putative genes related to sugar sensing process with *Arabidopsis thaliana* sequences in the NCBI database (http://www.ncbi.nlm.nih.gov) using Blastp analysis. (DOCX)

S5 Table Relative gene expression of sugar sensing and trehalose biosynthesis pathway associated genes of zygotic embryo stages (GZE, CZE, MZE, CZEMG and MZEMG) and two embryogenic cultures in proliferation (SE1 and SE6) and maturation (S1M and S6M) phase of *A. angustifolia*. Values are presented in average ± standard deviation.
(DOCX)

S1 Appendix The protein sequences are conserved among the key players in sugar-mediated metabolic status. (DOCX)
