# Supplementary material for: Carbohydrate-mediated responses during zygotic and early somatic embryogenesis in the endangered conifer, Araucaria angustifolia
Source: PLoS One. 2017 Jul 5;12(7):e0180051. doi: 10.1371/journal.pone.0180051 (PMC5497979; doi:10.1371/journal.pone.0180051)
Supplement: S1 Fig — Cell wall monosaccharides composition (%) of zygotic embryo stages–GZE (a), CZE (b), MZE (c), CZEMG (d) and MZEMG (e)–and two embryogenic cultures in proliferation–SE1 (f) and SE6 (g)–and maturation–S1M (h) and S6M (i)–phase of A. angustifolia. (DOCX) [file pone.0180051.s001.docx]

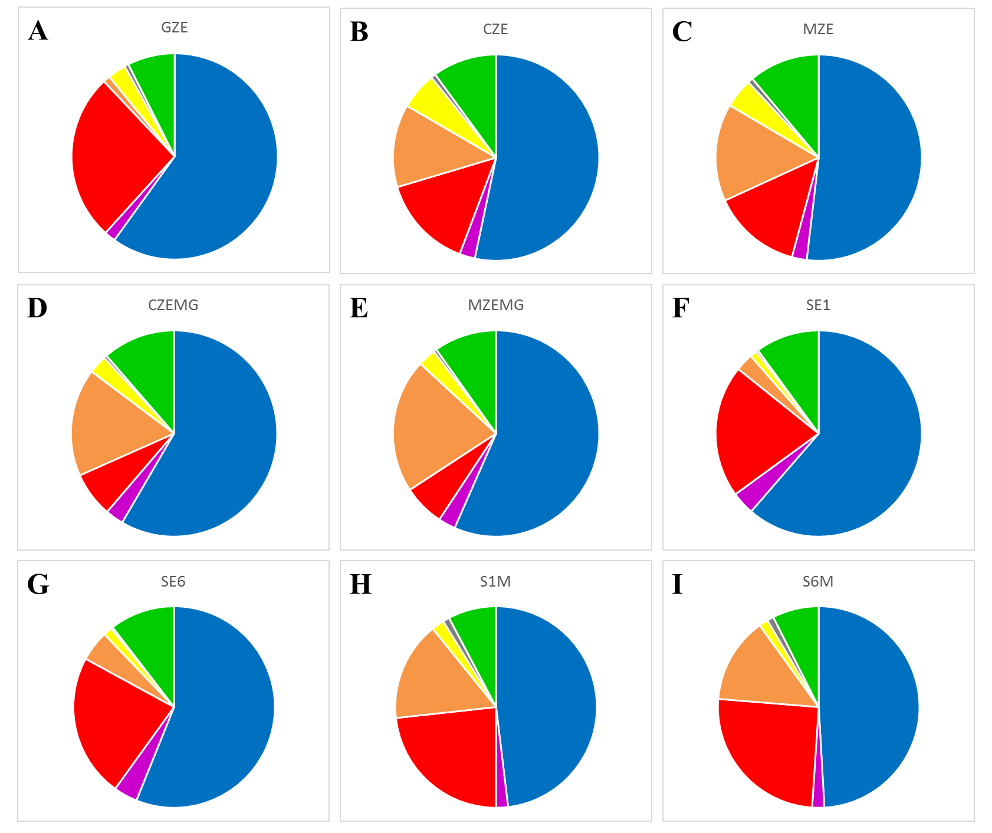


**Figure S1.** Cell wall monosaccharides composition (%) of zygotic embryo stages – GZE (a), CZE (b), MZE (c), CZEMG (d) and MZEMG (e) – and two embryogenic cultures in proliferation – SE1 (f) and SE6 (g) – and maturation – S1M (h) and S6M (i) – phase of *A. angustifolia*.
