# Supplementary material for: Carbohydrate-mediated responses during zygotic and early somatic embryogenesis in the endangered conifer, Araucaria angustifolia
Source: PLoS One. 2017 Jul 5;12(7):e0180051. doi: 10.1371/journal.pone.0180051 (PMC5497979; doi:10.1371/journal.pone.0180051)
Supplement: S2 Fig — Phylogenetic trees constructed from sequences with homology to Araucaria angustifolia TOR (a), RAPTOR (b), LST8 (c), SnRK1 (d), UGP (e), TPS (f) and TPP (g). For TPS and TPP, the phylogenetic trees were constructed based on previous studies of [91] and [81], respectively. The trees were built with the maximum likelihood method using PhyML program [48] based on a multiple sequence alignment generated by MEGA 6.0 [46]. The evolutionary mode was estimated applying JTT substitution model and the tree topology was performed by Subtree Pruning and Regrafting (SPR) and the branch support values was improved by approximate likelihood ratio test (aLRT). The colors green, light brown and red represents the Viridiplantae, Fungi and Animalia clades, respectively. Database and accession numbers are listed in S1 Table. (DOCX) [file pone.0180051.s002.docx]

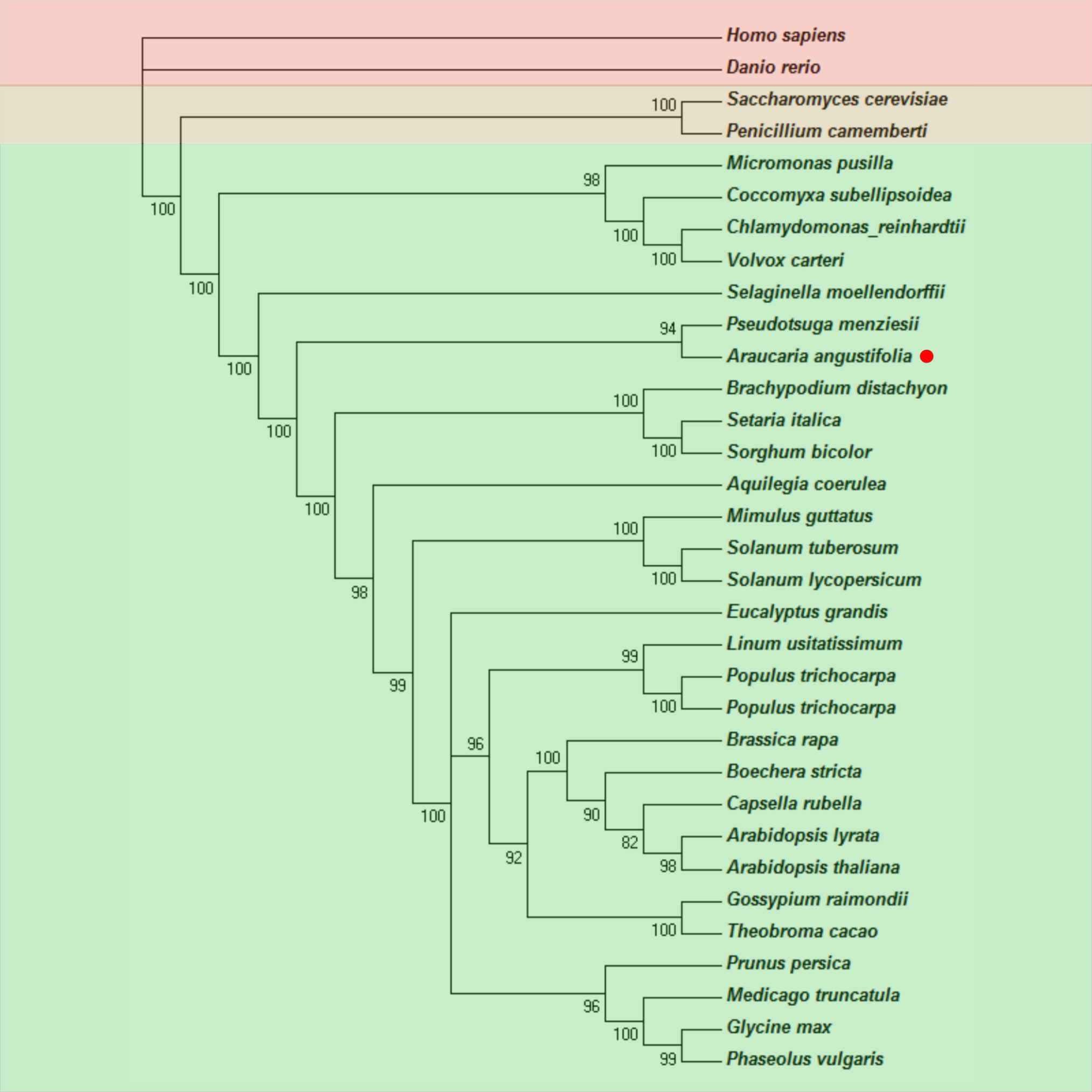


**A**


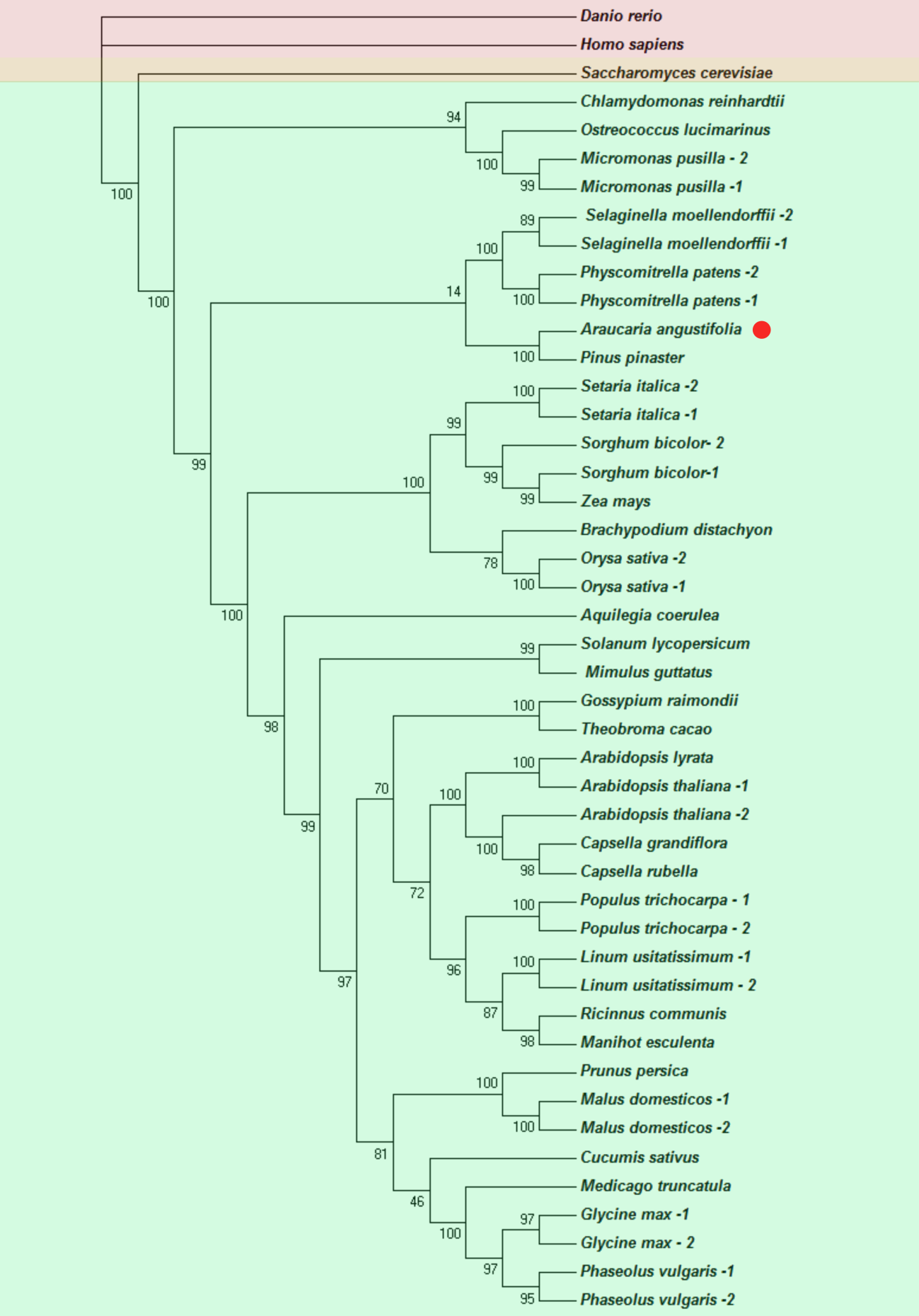


**B**


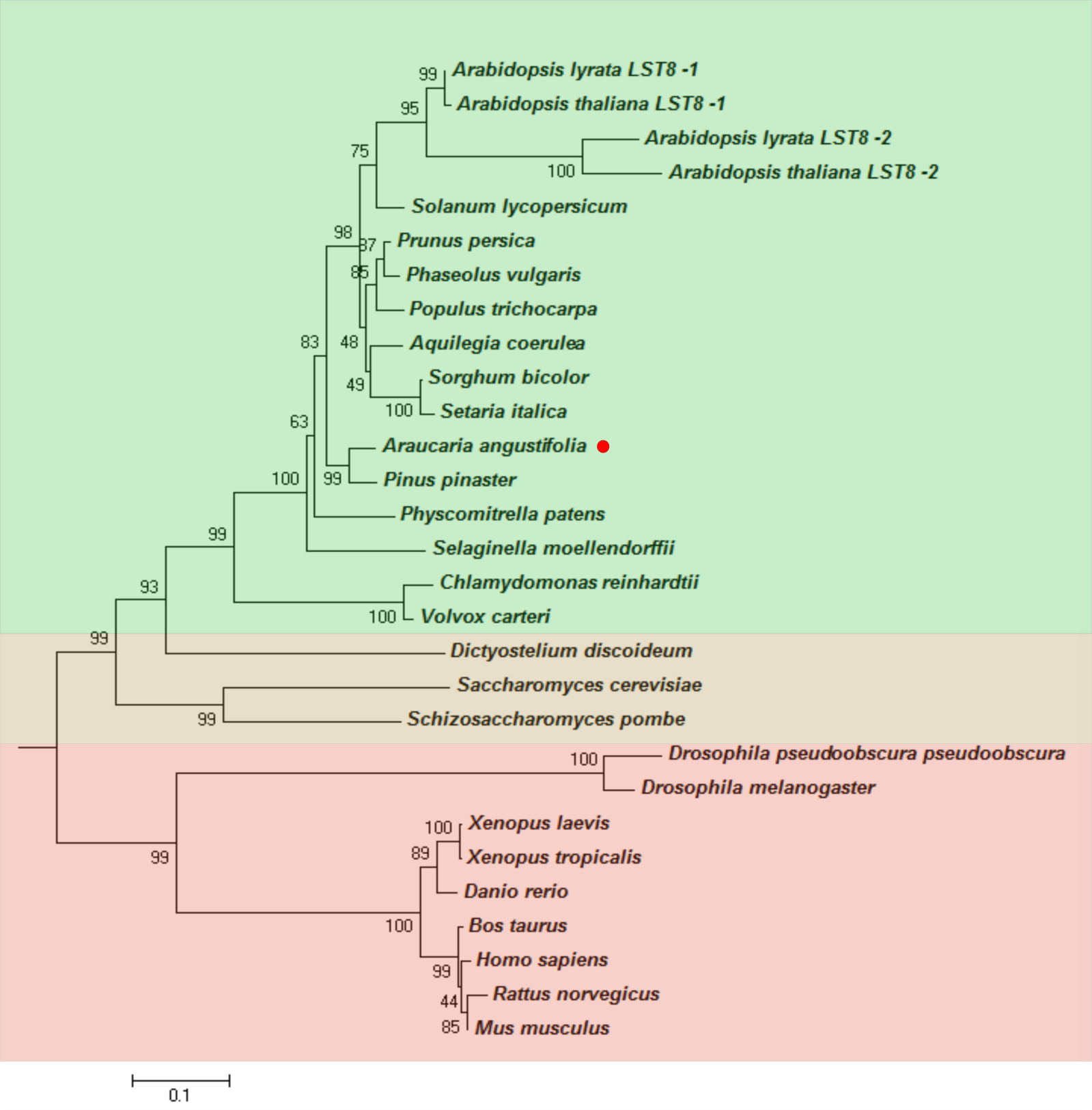


**C**


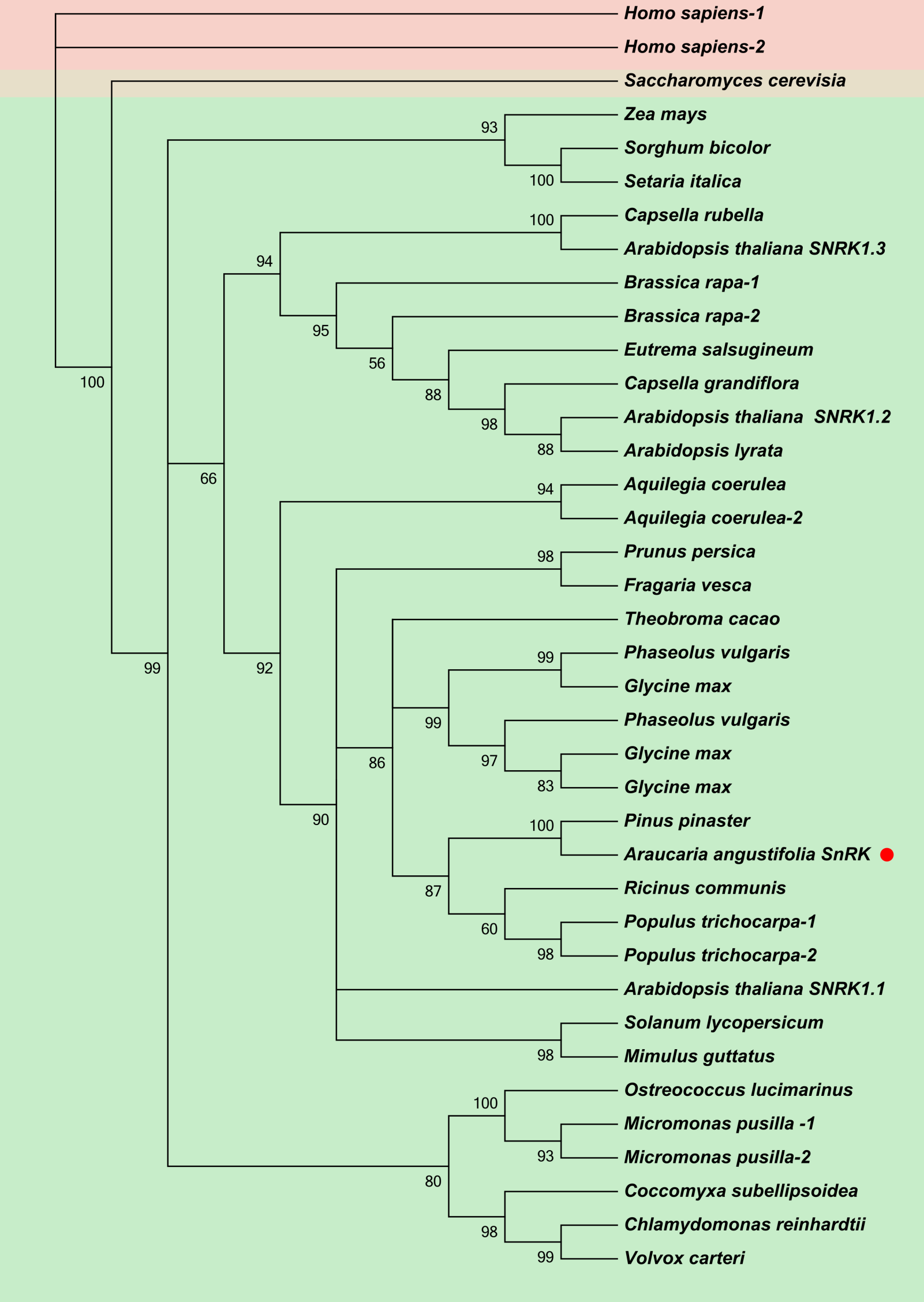

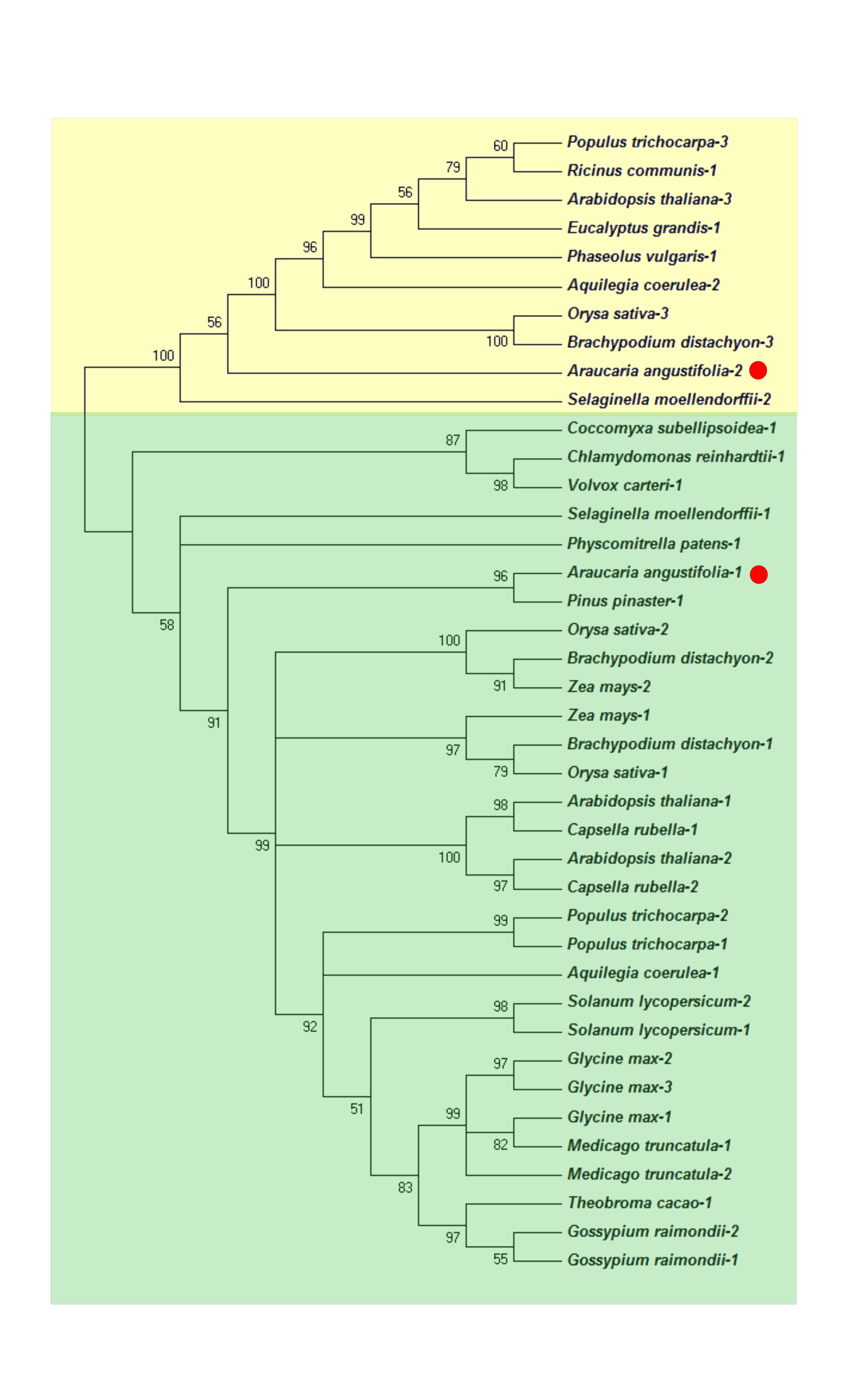


**D**

**E**


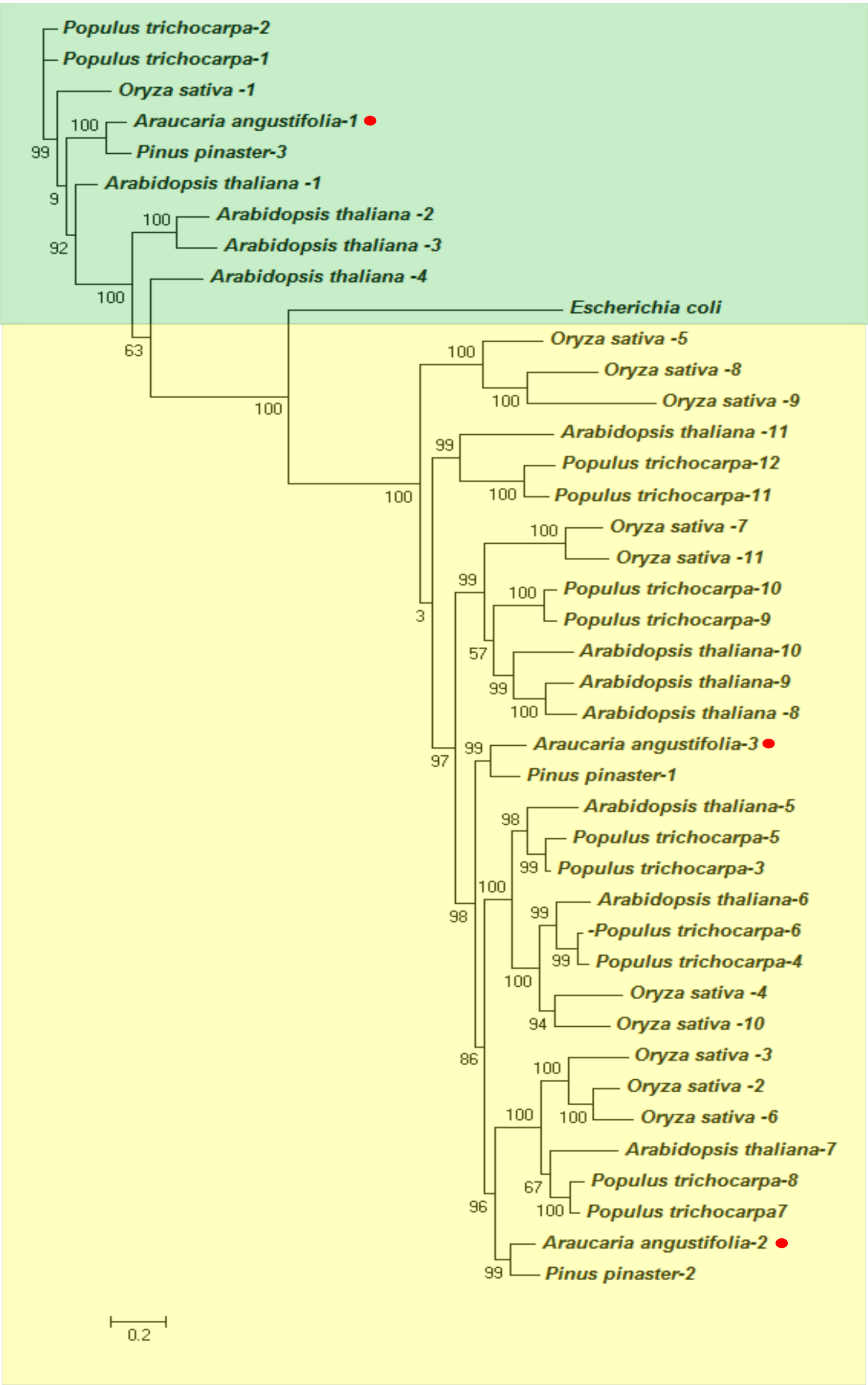


**F**


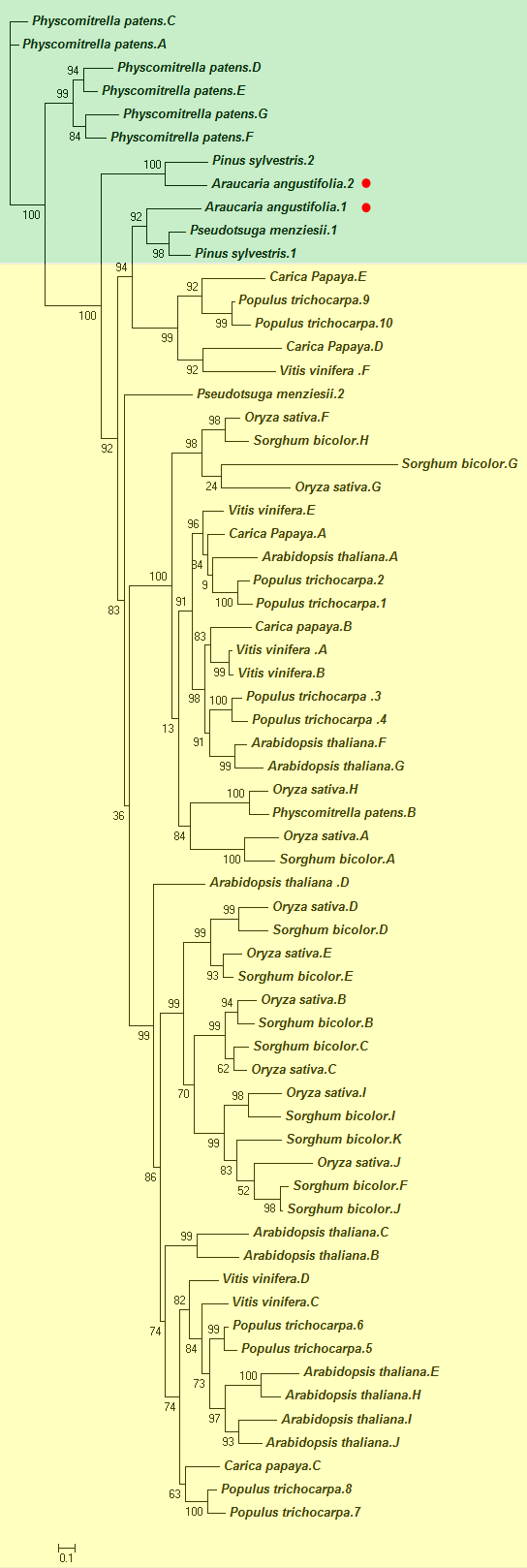


**G**

**Figure S2.** Phylogenetic trees constructed from sequences with homology to *Araucaria angustifolia* TOR (a), RAPTOR (b), LST8 (c), SnRK1 (d), UGP (e), TPS (f) and TPP (g). For TPS and TPP, the phylogenetic trees were constructed based on previous studies of [90] and [91], respectively. The trees were built with the maximum likelihood method using PhyML program [92] based on a multiple sequence alignment generated by MEGA 6.0 [46]. The evolutionary mode was estimated applying JTT substitution model and the tree topology was performed by Subtree Pruning and Regrafting (SPR) and the branch support values was improved by approximate likelihood ratio test (aLRT). The colors green, light brown and red represents the Viridiplantae, Fungi and Animalia clades, respectively. Database and accession numbers are listed in Table S1.
