# Supplementary material for: Carbohydrate-mediated responses during zygotic and early somatic embryogenesis in the endangered conifer, Araucaria angustifolia
Source: PLoS One. 2017 Jul 5;12(7):e0180051. doi: 10.1371/journal.pone.0180051 (PMC5497979; doi:10.1371/journal.pone.0180051)
Supplement: S3 Fig — TOR (a), RAPTOR (b), LST8 (c), SnRK1 (d), UGP (e), TPS (f) and TPP (g) domains multiple proteins sequences alignments. Shading indicates homology (black 90–100%, grey 70–90%) and species with accession numbers are available at S1 Table. (DOCX) [file pone.0180051.s003.docx]

**A**

**B**

**C**

**D**

**E**

**F**

**G**

**Figure S3.** TOR (a), RAPTOR (b), LST8 (c), SnRK1 (d), UGP (e), TPS (f) and TPP (g) domains multiple proteins sequences alignments. Shading indicates homology (black 90–100%, grey 70–90%) and species with accession numbers are available at Table S1.
