# Supplementary material for: Carbohydrate-mediated responses during zygotic and early somatic embryogenesis in the endangered conifer, Araucaria angustifolia
Source: PLoS One. 2017 Jul 5;12(7):e0180051. doi: 10.1371/journal.pone.0180051 (PMC5497979; doi:10.1371/journal.pone.0180051)
Supplement: S1 Table — (DOCX) [file pone.0180051.s004.docx]

**Table S1.** Sequences used for construction of phylogenetic trees

| Gene | Species | Locus | Database |
| --- | --- | --- | --- |
| TOR | *Aquilegia coerulea* | Aquca_015_00238.1 | Phytozome |
| TOR | *Arabidopsis lyrata* | 474140 | Phytozome |
| TOR | *Arabidopsis thaliana* | AT1G50030.1 | Phytozome |
| TOR | *Araucaria angustifolia* | comp54252_c0_seq14 | Araucaria |
| TOR | *Boechera stricta* | Bostr.26675s0164.1 | Phytozome |
| TOR | *Brachypodium distachyon* | Bradi2g28007.1 | Phytozome |
| TOR | *Brassica rapa* | Brara.F00328.1 | Phytozome |
| TOR | *Capsella rubella* | Carubv10008062m | Phytozome |
| TOR | *Chlamydomonas_reinhardtii* | Cre09.g400553.t1.1 | Phytozome |
| TOR | *Coccomyxa subellipsoidea* | 27394860 | Phytozome |
| TOR | *Danio rerio* | F1Q9H9 | UniProt |
| TOR | *Eucalyptus grandis* | Eucgr.I02503.1 | Phytozome |
| TOR | *Glycine max* | Glyma.01G241300.1 | Phytozome |
| TOR | *Gossypium raimondii* | Gorai.002G011200.1 | Phytozome |
| TOR | *Homo sapiens* | P42345 | UniProt |
| TOR | *Linum usitatissimum* | Lus10037246 | Phytozome |
| TOR | *Medicago truncatula* | Medtr5g005380.1 | Phytozome |
| TOR | *Micromonas pusilla* | e_gw2.16.279.1 | Phytozome |
| TOR | *Mimulus guttatus* | Migut.B00477.1 | Phytozome |
| TOR | *Penicillium camemberti* | A0A0G4NX55 | UniProt |
| TOR | *Phaseolus vulgaris* | Phvul.002G049900.1 | Phytozome |
| TOR | *Physcomitrella patens* | Phpat.006G017000 | Phytozome |
| TOR | *Pseudotsuga menziesii* | PME00006473 | PLAZA |
| TOR | *Populus trichocarpa* | Potri.001G289200.1 | Phytozome |
| TOR | *Populus trichocarpa* | Potri.009G084500.1 | Phytozome |
| TOR | *Prunus pérsica* | ppa000022m | Phytozome |
| TOR | *Saccharomyces cerevisiae* | AJS34492.1 | UniProt |
| TOR | *Selaginella moellendorffii* | fgenesh2_pg.C_scaffold_20000370 | Phytozome |
| TOR | *Setaria itálica* | Si020944m | Phytozome |
| TOR | *Solanum lycopersicum* | Solyc01g106770.2.1 | Phytozome |
| TOR | *Solanum tuberosum* | PGSC0003DMG401025733 | Phytozome |
| TOR | *Sorghum bicolor* | Sobic.009G109200.1 | Phytozome |
| TOR | *Theobroma cacao* | Thecc1EG007272t1 | Phytozome |
| TOR | *Volvox carteri* | Vocar20007444m | Phytozome |
| RAPTOR | *Aquilegia coerulea* | Aquca_005_00187.1 | Phytozome |
| RAPTOR | *Arabidopsis lyrata* | AT5G01770.1. | Phytozome |
| RAPTOR | *Arabidopsis thaliana -1* | AT3G08850.1 | Phytozome |
| RAPTOR | *Arabidopsis thaliana-2* | AT5G01770.1 | Phytozome |
| RAPTOR | *Araucaria angustifolia* | comp52312_c0_seq1 | Araucaria |
| RAPTOR | *Brachypodium distachyon* | Bradi4g44667.2 | Phytozome |
| RAPTOR | *Capsella grandiflora* | Cagra.2991s0009.1. | Phytozome |
| RAPTOR | *Capsella rubella* | Carubv10015752m | Phytozome |
| RAPTOR | *Chlamydomonas_reinhardtii* | Cre08.g371957 | Phytozome |
| RAPTOR | *Cucumis sativus* | Cucsa.130350.1 | Phytozome |
| RAPTOR | *Danio rerio* | tr\|A0A0R4IFI0\| | Uniprot |
| RAPTOR | *Glycine max -1* | Glyma.18G210300.1 | Phytozome |
| RAPTOR | *Glycine max-2* | Glyma.09G278500.1 | Phytozome |
| RAPTOR | *Gossypium raimondii* | Gorai.005G006600.1 | Phytozome |
| RAPTOR | *Gossypium raimondii* | Gorai.005G006600.1. | Phytozome |
| RAPTOR | *Homo sapiens* | sp\|Q8N122 | Uniprot |
| RAPTOR | *Linum usitatissimum* | Lus10009083 | Phytozome |
| RAPTOR | *Linum usitatissimum* | Lus10025263 | Phytozome |
| RAPTOR | *Malus domesticos -1* | MDP0000061330 | Phytozome |
| RAPTOR | *Malus domesticos-2* | MDP0000061332 | Phytozome |
| RAPTOR | *Manihot esculenta* | cassava4.1_000302m | Phytozome |
| RAPTOR | *Medicago truncatula* | Medtr7g072330.1 | Phytozome |
| RAPTOR | *Micromonas pusilla -1* | 17707.mpu.27348339 | Phytozome |
| RAPTOR | *Micromonas pusilla -2* | 80694.mpu.27400538 | Phytozome |
| RAPTOR | *Mimulus guttatus* | Migut.J00073.1 | Phytozome |
| RAPTOR | *Orysa sativa -1* | LOC_Os11g01872.1 | Phytozome |
| RAPTOR | *Orysa sativa -2* | LOC_Os12g01922.1 | Phytozome |
| RAPTOR | *Ostreococcus lucimarinus* | 41144.olu.27418578 | Phytozome |
| RAPTOR | *Phaseolus vulgaris -1* | Phvul.008G087800.1 | Phytozome |
| RAPTOR | *Phaseolus vulgaris -2* | Phvul.008G088100.1 | Phytozome |
| RAPTOR | *Physcomitrella patens* | Phpat.017G012600.1 | Phytozome |
| RAPTOR | *Physcomitrella patens* | Phpat.025G027400.1 | Phytozome |
| RAPTOR | *Pinus pinaster* | ppi_RAPTOR_sp_v3.0_unigene2351 | Sustain Pine |
| RAPTOR | *Populus trichocarpa - 1* | Potri.006G106600.1 | Phytozome |
| RAPTOR | *Populus trichocarpa 2* | Potri.016G132000.1 | Phytozome |
| RAPTOR | *Prunus persica* | ppa000282m | Phytozome |
| RAPTOR | *Ricinnus communis* | 29718.m000133 | Phytozome |
| RAPTOR | *Saccharomyces cerevisiae* | \|P38873.2 | Uniprot |
| RAPTOR | *Selaginella moellendorffii* | 171199 (M2) PTHR12848:SF16 | Phytozome |
| RAPTOR | *Selaginella moellendorffii* | 171199 (M2) PTHR12848:SF16 | Phytozome |
| RAPTOR | *Setaria italica -1* | Si025822m | Phytozome |
| RAPTOR | *Setaria italica -2* | Si009187m | Phytozome |
| RAPTOR | *Solanum lycopersicum* | Solyc09g014780.2. | Phytozome |
| RAPTOR | *Sorghum bicolor* | Sobic.008G009700.1 | Phytozome |
| RAPTOR | *Sorghum bicolor-1* | Sobic.005G008800.1. | Phytozome |
| RAPTOR | *Theobroma cacao* | Thecc1EG019767t1 | Phytozome |
| RAPTOR | *Zea mays* | GRMZM2G048067_P01 | Phytozome |
| LST8 | *Aquilegia coerulea* | Aquca_003_00043.1 | Phytozome |
| LST8 | *Arabidopsis lyrata* | fgenesh1_pg.C_scaffold_4000102 | Phytozome |
| LST8 | *Arabidopsis lyrata* | fgenesh2_kg.3__2005__AT3G18140.1 | Phytozome |
| LST8 | *Arabidopsis thaliana* | AT2G22040.1 | Phytozome |
| LST8 | *Arabidopsis thaliana* | AT3G18140.1 | Phytozome |
| LST8 | *Araucaria angustifolia* | comp36842_c0_seq1 | Araucaria |
| LST8 | *Chlamydomonas_reinhardtii* | Cre17.g713900.t1.2 | Phytozome |
| LST8 | *Phaseolus vulgaris* | Phvul.006G173700.1 | Phytozome |
| LST8 | *Physcomitrella patens* | Phpat.011G100100.1.p | Phytozome |
| LST8 | *Pinus pinaster* | unigene2607 | Sustain Pine |
| LST8 | *Populus trichocarpa* | Potri.016G052800.1 | Phytozome |
| LST8 | *Prunus persica* | Prupe.7G067800 | Phytozome |
| LST8 | *Selaginella moellendorffii* | fgenesh1_pm.C_scaffold_52000028 | Phytozome |
| LST8 | *Setaria italica* | Si036688m | Phytozome |
| LST8 | *Solanum lycopersicum* | Solyc03g059310.2.1 | Phytozome |
| LST8 | *Sorghum bicolor* | Sobic.005G172500.1. | Phytozome |
| LST8 | *Volvox carteri* | Vocar20015220m | Phytozome |
| LST8 | *Dictyostelium discoideum* | Q54D08 | Uniprot |
| LST8 | *Danio rerio* | Q803V5 | Uniprot |
| LST8 | *Homo sapiens* | Q9BVC4 | Uniprot |
| LST8 | *Rattus norvegicus* | Q9Z2K5 | Uniprot |
| LST8 | *Bos taurus* | Q17QU5 | Uniprot |
| LST8 | *Mus musculus* | Q9DCJ1 | Uniprot |
| LST8 | *Xenopus laevis* | Q6PA72 | Uniprot |
| LST8 | *Xenopus tropicalis* | Q5I0B4 | Uniprot |
| LST8 | *Saccharomyces cerevisiae* | P41318 | Uniprot |
| LST8 | *Schizosaccharomyces pombe* | O74184 | Uniprot |
| LST8 | *Drosophila pseudoobscura* | Q29HG9 | Uniprot |
| LST8 | *Drosophila melanogaster* | Q9W328 | Uniprot |
| SnRK1 | *Aquilegia coerulea* | Aquca03500179.1 | Phytozome |
| SnRK1 | *Aquilegia coerulea* | Aquca00200708.1 | Phytozome |
| SnRK1 | *Arabidopsis lyrata* | fgenesh2kg.5675AT3G29160.2 | Phytozome |
| SnRK1 | *Arabidopsis thaliana* | AT5G39440.1 | Phytozome |
| SnRK1 | *Arabidopsis thaliana* | AT3G01090.2 | Phytozome |
| SnRK1 | *Arabidopsis thaliana* | AT3G29160.1 | Phytozome |
| SnRK1 | *Araucaria angustifolia* | comp53145_c0_seq1 | Phytozome |
| SnRK1 | *Brassica rapa* | Brara.F03101.1 | Phytozome |
| SnRK1 | *Brassica rapa* | Brara.B03362.1 | Phytozome |
| SnRK1 | *Capsella grandiflora* | Cagra.18325s0001.1 | Phytozome |
| SnRK1 | *Capsella rubella* | Carubv10006326m | Phytozome |
| SnRK1 | *Chlamydomonas_reinhardtii* | Cre04.g211600.t1.1 | Phytozome |
| SnRK1 | *Coccomyxa subellipsoidea* | e_gw1.10.111.1 | Phytozome |
| SnRK1 | *Eutrema salsugineum* | Thhalv10004026m | Phytozome |
| SnRK1 | *Fragaria vesca* | mrna04397.1-v1.0-hybrid | Phytozome |
| SnRK1 | *Glycine max* | Glyma.18G262900.1. | Phytozome |
| SnRK1 | *Glycine max* | Glyma.08G240300.1. | Phytozome |
| SnRK1 | *Glycine max* | Glyma.13G060400.1 | Phytozome |
| SnRK1 | *Homo sapiens* | sp\|P54646\|AAPK2HUMAN | Uniprot |
| SnRK1 | *Homo sapiens* | sp\|Q13131\|AAPK1HUMAN | Uniprot |
| SnRK1 | *Micromonas pusilla* | MicpuC2.estExt_Genewise1Plus.C_110349 | Phytozome |
| SnRK1 | *Mimulus guttatus* | Migut.H00673.1 | Phytozome |
| SnRK1 | *Ostreococcus lucimarinus* | fgenesh1_pm.C_Chr_6000070 | Phytozome |
| SnRK1 | *Phaseolus vulgaris* | Phvul.008G039400.1 | Phytozome |
| SnRK1 | *Phaseolus vulgaris* | Phvul.004G032000.1 | Phytozome |
| SnRK1 | *Pinus pinaster* | unigene3364 | SustainPine |
| SnRK1 | *Populus trichocarpa* | Potri.017G087900.1 | Phytozome |
| SnRK1 | *Populus trichocarpa* | Potri.004G115900.1 | Phytozome |
| SnRK1 | *Prunus persica* | Prupe.3G262900 | Phytozome |
| SnRK1 | *Ricinus communis* | 29635.m000462 | Phytozome |
| SnRK1 | *Saccharomyces cerevisiae* | sp\|P06782\|SNF1 YEAST | Uniprot |
| SnRK1 | *Setaria italica* | Seita.9G442000 | Phytozome |
| SnRK1 | *Solanum lycopersicum* | Solyc02g067030.2.1 | Phytozome |
| SnRK1 | *Sorghum bicolor* | Sobic.001G408200.1 | Phytozome |
| SnRK1 | *Theobroma cacao* | Thecc1EG018660 | Phytozome |
| SnRK1 | *Volvox carteri* | Vocar20002008m | Phytozome |
| SnRK1 | *Zea mays* | GRMZM2G077278 | Phytozome |
| UGP | *Aquilegia coerulea-1* | Aquca_002_01330 | Phytozome |
| UGP | *Aquilegia coerulea-2* | Aquca_045_00200 | Phytozome |
| UGP | *Arabidopsis thaliana-1* | AT3G03250 | Phytozome |
| UGP | *Arabidopsis thaliana-2* | AT5G17310 | Phytozome |
| UGP | *Arabidopsis thaliana-3* | AT3G56040 | Phytozome |
| UGP | *Araucaria angustifolia-1* | comp39733_c0_seq1 | Araucaria |
| UGP | *Araucaria angustifolia-2* | comp50296_c0_seq4 | Araucaria |
| UGP | *Brachypodium distachyon-1* | Bradi4g37350 | Phytozome |
| UGP | *Brachypodium distachyon-2* | Bradi3g01640 | Phytozome |
| UGP | *Brachypodium distachyon-3* | Bradi2g22680 | Phytozome |
| UGP | *Capsella rubella-1* | Carubv10013624m | Phytozome |
| UGP | *Capsella rubella-2* | Carubv10000883m | Phytozome |
| UGP | *Chlamydomonas reinhardtii-1* | Cre04.g229700.t1 | Phytozome |
| UGP | *Coccomyxa subellipsoidea-1* | 27385894 | Phytozome |
| UGP | *Eucalyptus grandis-1* | Eucgr.J00183 | Phytozome |
| UGP | *Glycine max-1* | Glyma.13G152500 | Phytozome |
| UGP | *Glycine max-2* | Glyma.02G241100 | Phytozome |
| UGP | *Glycine max-3* | Glyma.14G210700 | Phytozome |
| UGP | *Gossypium raimondii-1* | Gorai.004G048300 | Phytozome |
| UGP | *Gossypium raimondii-2* | Gorai.007G188400 | Phytozome |
| UGP | *Medicago truncatula-1* | Medtr3g064490 | Phytozome |
| UGP | *Medicago truncatula-2* | Medtr5g077000 | Phytozome |
| UGP | *Orysa sativa-1* | LOC_Os09g38030 | Phytozome |
| UGP | *Orysa sativa-2* | LOC_Os02g02560 | Phytozome |
| UGP | *Orysa sativa-3* | LOC_Os05g39230 | Phytozome |
| UGP | *Phaseolus vulgaris-1* | Phvul.001G244900 | Phytozome |
| UGP | *Physcomitrella patens-1* | Phpat.001G001100 | Phytozome |
| UGP | *Pinus pinaster-1* | unigene11864 | Sustainpine |
| UGP | *Populus trichocarpa-1* | Potri.017G144700 | Phytozome |
| UGP | *Populus trichocarpa-2* | Potri.004G074400 | Phytozome |
| UGP | *Populus trichocarpa-3* | Potri.010G185800 | Phytozome |
| UGP | *Ricinus communis-1* | 29814.t000031 | Phytozome |
| UGP | *Selaginella moellendorffii-1* | 15417374 | Phytozome |
| UGP | *Selaginella moellendorffii-2* | 150184 | Phytozome |
| UGP | *Solanum lycopersicum-1* | Solyc11g011960 | Phytozome |
| UGP | *Solanum lycopersicum-2* | Solyc05g054060 | Phytozome |
| UGP | *Theobroma cacao-1* | Thecc1EG016775t1 | Phytozome |
| UGP | *Volvox carteri-1* | Vocar20009812m | Phytozome |
| UGP | *Zea mays-1* | GRMZM2G032003 | Phytozome |
| UGP | *Zea mays-2* | GRMZM2G098370 | Phytozome |
| TPS | *Arabidopsis thaliana -1* | At1g78580 | Phytozome |
| TPS | *Arabidopsis thaliana -2* | At1g16980 | Phytozome |
| TPS | *Arabidopsis thaliana -3* | At1g17000 | Phytozome |
| TPS | *Arabidopsis thaliana -4* | At4g27550 | Phytozome |
| TPS | *Arabidopsis thaliana -5* | At4g17770 | Phytozome |
| TPS | *Arabidopsis thaliana -6* | At1g68020 | Phytozome |
| TPS | *Arabidopsis thaliana -7* | At1g06410 | Phytozome |
| TPS | *Arabidopsis thaliana -8* | At1g70290 | Phytozome |
| TPS | *Arabidopsis thaliana -9* | At1g23870 | Phytozome |
| TPS | *Arabidopsis thaliana -10* | At1g60140 | Phytozome |
| TPS | *Arabidopsis thaliana -11* | At2g18700 | Phytozome |
| TPS | *Araucaria angustifoli-1* | comp46652_c0_seq2 | Araucaria |
| TPS | *Araucaria angustifoli-2* | comp50204_c0_seq1 | Araucaria |
| TPS | *Araucaria angustifoli-3* | comp52170_c0_seq5 | Araucaria |
| TPS | *Oryza sativa -1* | LOC_Os05g44210 | Phytozome |
| TPS | *Oryza sativa -2* | LOC_Os01g54560 | Phytozome |
| TPS | *Oryza sativa -3* | LOC_Os01g53000 | Phytozome |
| TPS | *Oryza sativa -4* | LOC_Os03g12360 | Phytozome |
| TPS | *Oryza sativa -5* | LOC_Os02g54820 | Phytozome |
| TPS | *Oryza sativa -6* | LOC_Os05g44100 | Phytozome |
| TPS | *Oryza sativa -7* | LOC_Os08g31980 | Phytozome |
| TPS | *Oryza sativa -8* | LOC_Os08g34580 | Phytozome |
| TPS | *Oryza sativa -9* | LOC_Os09g25890 | Phytozome |
| TPS | *Oryza sativa -10* | LOC_Os09g23350 | Phytozome |
| TPS | *Oryza sativa -11* | LOC_Os09g20990 | Phytozome |
| TPS | *Populus trichocarpa -1* | estExt_fgenesh4_pg.C_1680018 | JGI Populus trichocarpa v1.1 |
| TPS | *Populus trichocarpa -2* | e_gw1.IV.2524.1 | JGI Populus trichocarpa v1.1 |
| TPS | *Populus trichocarpa -3* | fgenesh4_pg.C_LG_III000738 | JGI Populus trichocarpa v1.1 |
| TPS | *Populus trichocarpa -4* | grail3.0010065002 | JGI Populus trichocarpa v1.1 |
| TPS | *Populus trichocarpa -5* | estExt_Genewise1_v1.C_290287 | JGI Populus trichocarpa v1.1 |
| TPS | *Populus trichocarpa -6* | estExt_Genewise1_v1.C_LG_X6311 | JGI Populus trichocarpa v1.1 |
| TPS | *Populus trichocarpa -7* | eugene3.00110684 | JGI Populus trichocarpa v1.1 |
| TPS | *Populus trichocarpa -8* | fgenesh4_pg.C_LG_IV000367 | JGI Populus trichocarpa v1.1 |
| TPS | *Populus trichocarpa -9* | fgenesh4_pm.C_LG_XII000278 | JGI Populus trichocarpa v1.1 |
| TPS | *Populus trichocarpa -10* | eugene3.00150531 | JGI Populus trichocarpa v1.1 |
| TPS | *Populus trichocarpa -11* | eugene3.00061363 | JGI Populus trichocarpa v1.1 |
| TPS | *Populus trichocarpa -12* | fgenesh4_pm.C_LG_XVIII000320 | JGI Populus trichocarpa v1.1 |
| TPS | *Escherichia coli* | sp\|P31677\|OTSA_ECOLI | Uniprot |
| TPP | *Arabidopsis thaliana -A* | AT5G51460 | PLAZA |
| TPP | *Arabidopsis thaliana -B* | AT1G78090 | PLAZA |
| TPP | *Arabidopsis thaliana –C* | AT1G22210 | PLAZA |
| TPP | *Arabidopsis thaliana –D* | AT1G35910 | PLAZA |
| TPP | *Arabidopsis thaliana –E* | AT2G22190 | PLAZA |
| TPP | *Arabidopsis thaliana –F* | AT4G12430 | PLAZA |
| TPP | *Arabidopsis thaliana –G* | AT4G22590 | PLAZA |
| TPP | *Arabidopsis thaliana –H* | AT4G39770 | PLAZA |
| TPP | *Arabidopsis thaliana –I* | AT5G10100 | PLAZA |
| TPP | *Arabidopsis thaliana –J* | AT5G65140 | PLAZA |
| TPP | *Araucaria angustifolia -1* | comp50128_c0_seq1 | Araucaria |
| TPP | *Araucaria angustifolia -2* | comp117996_c0_seq1 | Araucaria |
| TPP | *Carica Papaya –A* | CP00003G03160 | PLAZA |
| TPP | *Carica Papaya –B* | CP00029G01250 | PLAZA |
| TPP | *Carica Papaya –C* | CP00036G01120 | PLAZA |
| TPP | *Carica Papaya –D* | CP00273G00020 | PLAZA |
| TPP | *Carica Papaya –E* | CP00405G00050 | PLAZA |
| TPP | *Oryza sativa –A* | OS07G30160 | PLAZA |
| TPP | *Oryza sativa –B* | OS09G20390 | PLAZA |
| TPP | *Oryza sativa –C* | OS08G31630 | PLAZA |
| TPP | *Oryza sativa –D* | OS06G11840 | PLAZA |
| TPP | *Oryza sativa –E* | OS02G51680 | PLAZA |
| TPP | *Oryza sativa –F* | OS02G44230 | PLAZA |
| TPP | *Oryza sativa –G* | OS04G46760 | PLAZA |
| TPP | *Oryza sativa –H* | OS10G40550 | PLAZA |
| TPP | *Oryza sativa –I* | OS03G26910 | PLAZA |
| TPP | *Oryza sativa –J* | OS07G43160 | PLAZA |
| TPP | *Physcomitrella patens –A* | PP00201G00540 | PLAZA |
| TPP | *Physcomitrella patens –B* | PP00141G00660 | PLAZA |
| TPP | *Physcomitrella patens –C* | PP00047G00570 | PLAZA |
| TPP | *Physcomitrella patens –D* | PP00032G01010 | PLAZA |
| TPP | *Physcomitrella patens –E* | PP00333G00270 | PLAZA |
| TPP | *Physcomitrella patens –F* | PP00046G00150 | PLAZA |
| TPP | *Physcomitrella patens –G* | PP00227G00210 | PLAZA |
| TPP | *Pinus sylvestris -1* | PSY00012422 | PLAZA |
| TPP | *Pinus sylvestris -2* | PSY00019014 | PLAZA |
| TPP | *Populus trichocarpa -1* | PT15G09950 | PLAZA |
| TPP | *Populus trichocarpa -2* | PT12G12350 | PLAZA |
| TPP | *Populus trichocarpa -3* | PT00G20150 | PLAZA |
| TPP | *Populus trichocarpa -4* | PT03G08130 | PLAZA |
| TPP | *Populus trichocarpa -5* | PT07G05290 | PLAZA |
| TPP | *Populus trichocarpa -6* | PT05G01760 | PLAZA |
| TPP | *Populus trichocarpa -7* | PT05G07710 | PLAZA |
| TPP | *Populus trichocarpa -8* | PT02G08940 | PLAZA |
| TPP | *Populus trichocarpa -9* | PT12G01420 | PLAZA |
| TPP | *Populus trichocarpa -10* | PT15G02080 | PLAZA |
| TPP | *Pseudotsuga menziesii -1* | PME00091865 | PLAZA |
| TPP | *Pseudotsuga menziesii -2* | PME00022560 | PLAZA |
| TPP | *Sorghum bicolor –A* | SB02G033420 | PLAZA |
| TPP | *Sorghum bicolor –B* | SB02G023260 | PLAZA |
| TPP | *Sorghum bicolor –C* | SB07G020100 | PLAZA |
| TPP | *Sorghum bicolor –D* | SB10G007770 | PLAZA |
| TPP | *Sorghum bicolor –E* | SB04G027650 | PLAZA |
| TPP | *Sorghum bicolor –F* | SB02G039790 | PLAZA |
| TPP | *Sorghum bicolor –G* | SB08G006160 | PLAZA |
| TPP | *Sorghum bicolor –H* | SB01G029590 | PLAZA |
| TPP | *Sorghum bicolor –I* | SB01G033800 | PLAZA |
| TPP | *Sorghum bicolor –J* | SB02G039810 | PLAZA |
| TPP | *Sorghum bicolor –K* | SB02G039820 | PLAZA |
| TPP | *Vitis vinifera –A* | VV00G38520 | PLAZA |
| TPP | *Vitis vinifera –B* | VV02G05800 | PLAZA |
| TPP | *Vitis vinifera –C* | VV00G54490 | PLAZA |
| TPP | *Vitis vinifera –D* | VV18G04460 | PLAZA |
| TPP | *Vitis vinifera –E* | VV00G11435 | PLAZA |
| TPP | *Vitis vinifera –F* | VV00G60285 | PLAZA |
