# Supplementary material for: Carbohydrate-mediated responses during zygotic and early somatic embryogenesis in the endangered conifer, Araucaria angustifolia
Source: PLoS One. 2017 Jul 5;12(7):e0180051. doi: 10.1371/journal.pone.0180051 (PMC5497979; doi:10.1371/journal.pone.0180051)
Supplement: S2 Table — (DOCX) [file pone.0180051.s005.docx]

**Table S2.** List of primer sequences used in the qPCR analysis of the sugar sensing associated candidate genes.

| **Gene** | **Primer sequences (forward/reverse primer)** | **Expected amplicon size (bp)** |
| --- | --- | --- |
| *AaTOR* | 5'-GAGCCACTTGTTTTAGAGTCCAG3' / 5'-CAATCTTCCTTCGTACTTCGTTGAG-3' | 206 |
| *AaRAPTOR* | 5'-GCCTCTTCTATCTGAACCTC-3' / 5'-CAGCCCTAACAAGTGGACTC-3' | 203 |
| *AaLST8* | 5'-GAAGAAGCAGTCACCAAG-3' / 5'-CTCCAAGCACATAACACATAC-3' | 212 |
| *AaSnRK1* | 5'-GGTCATCCAGTTCCAACGCCG-3' / 5'-GTAATGTCCTATCTTCTTCCATCG-3' | 195 |
| *AaUGP1* | 5'-GAAGTTGTGGTTCCCTATGC-3' / 5'-CTCTGCTATTGTATTTGTCGTTGAG-3' | 214 |
| *AaTPS1* | 5'-GCACACTCCCTTCCCTTCTTC-3' / 5'-CTTCCCTTGGTCCTCTACTC-3' | 187 |
| *AaTPS2* | 5'-GGAGGGGCAAAGTTGTCTTG-3' / 5'-CAGTAACCACAGCACATTCAGC-3' | 216 |
| *AaTPS3* | 5'-CGATGAATGTAGCCCTCACTATGC-3' / 5'-CTCAATCCAAATCCAATACCCCAGC-3' | 178 |
| *AaTPP1* | 5'-CTCATCTAAGCCCACAGC-3' / 5'-CATTATGTCATCACAACTAGGTCC-3' | 196 |
| *AaTPP2* | 5'-CCACAAGGTCAAAACACAAG-3' / 5'-CCTCATAACATCTAGCCACTC-3' | 229 |
