# Supplementary material for: Carbohydrate-mediated responses during zygotic and early somatic embryogenesis in the endangered conifer, Araucaria angustifolia
Source: PLoS One. 2017 Jul 5;12(7):e0180051. doi: 10.1371/journal.pone.0180051 (PMC5497979; doi:10.1371/journal.pone.0180051)
Supplement: S3 Table — Values are presented in average ± standard error. (DOCX) [file pone.0180051.s006.docx]

**Table S3.** Non-Structural Carbohydrates (NSC) content (µg.mg^-1^ dry weight) of zygotic embryo stages (GZE, CZE, MZE, CZEMG and MZEMG) and two embryogenic cultures in proliferation (SE1 and SE6) and maturation (S1M and S6M) phase of *A. angustifolia*. Values are presented in average ± standard error.

| *Samples* | *Glucose* | *Fructose* | *Sucrose* | *Raffinose* | *Myo-inositol* | *Starch* | *Sucrose : Hexose ratio* |
| --- | --- | --- | --- | --- | --- | --- | --- |
| *GZE* | 213.28 ± 1.91 A | 209.37 ± 1.61 A | 4.75 ± 0.14 E | n.d. | 4.54 ± 0.09 C | 1.74 ± 0.20 E | 0.01 ± 0.00 C |
| *CZE* | 3.85 ± 0.27 D | 4.52 ± 0.10 DE | 50.06 ± 8.37 B | 5.82 ± 0.88 B | 8.31 ± 1.51 AB | 304.82 ± 26.06 B | 5.91 ± 0.76 B |
| *MZE* | 2.85 ± 0.66 E | 6.41 ± 0.74 CD | 103.81 ± 5.01 A | 17.62 ± 1.72 A | 9.44 ± 0.59 AB | 315.88 ± 25.73 B | 11.49 ± 0.99 A |
| *CZEMG* | 3.04 ± 0.15 DE | 3.44 ± 0.18 E | 38.83 ± 7.80 CD | 2.86 ± 0.93 BC | 2.47 ± 0.21 D | 451.38 ± 7.85 A | 5.96 ± 1.08 B |
| *MZEMG* | 3.73 ± 0.30 D | 4.66 ± 0.55 DE | 30.75 ± 1.06 D | 1.31 ± 0.40 C | 2.22 ± 0.19 D | 314.28 ± 4.47 B | 3.72 ± 0.26 B |
| *SE1* | 281.01 ± 23.01 A | 206.15 ± 21.97 A | n.d. | n.d. | 13.40 ± 1.58 A | 45.90 ± 2.84 D | 0.00 ± 0.00 C |
| *SE6* | 117.94 ± 10.81 B | 41.94 ± 2.97 B | 75.06 ± 12.10 A | n.d. | 8.75 ± 1.24 AB | 127.56 ± 9.41 C | 0.46 ± 0.04 C |
| *S1M* | 126.15 ± 3.30 B | 7.01 ± 0.67 C | 53.96 ± 1.64 B | 2.00 ± 0.69 BC | 7.44 ± 0.24 BC | 138.18 ± 7.40 C | 0.41 ± 0.00 C |
| *S6M* | 71.90 ± 1.62 C | 8.51 ± 0.37 C | 42.50 ± 0.55 BC | 2.70 ± 0.30 BC | 7.45 ± 0.21 BC | 58.24 ± 4.35 D | 0.53 ± 0.01 C |

^a^ Samples according to Figure 1.
