# Supplementary material for: Carbohydrate-mediated responses during zygotic and early somatic embryogenesis in the endangered conifer, Araucaria angustifolia
Source: PLoS One. 2017 Jul 5;12(7):e0180051. doi: 10.1371/journal.pone.0180051 (PMC5497979; doi:10.1371/journal.pone.0180051)
Supplement: S4 Table — (DOCX) [file pone.0180051.s007.docx]

**Table S4.** Comparison of *Araucaria angustifolia* putative genes related to Sugar Sensing process with *Arabidopsis thaliana* sequences in the NCBI database (http://www.ncbi.nlm.nih.gov) using Blastp analysis.

| ***Gene*** | **A. angustifolia *protein length (aa)*** | ***BLASTp*** | ***Identities (%)*** | ***E-value*** | ***Bit score*** |
| --- | --- | --- | --- | --- | --- |
| *AaTOR* | 1856 | *A. thaliana* AT1G50030.1 TOR \| target of rapamycin | 71 | 0 | 2716 |
| *AaRAPTOR* | 1375 | *A. thaliana* AT3G08850.1 RAPTOR1B, ATRAPTOR1B, RAPTOR1 \| HEAT repeat ;WD domain, G-beta repeat protein | 61 | 0 | 1653 |
| *AaLST8* | 316 | *A. thaliana* AT3G18140.1 LETHAL WITH SEC THIRTEEN 8-1, LST8-1 | 83 | 1e^-156^ | 547 |
| *AaSnRK1* | 511 | *A. thaliana* AT3G01090.2 SNF1-RELATED PROTEIN KINASE 1.1, SNRK1.1 | 80 | 0 | 807 |
| *AaUGP1* | 477 | *A. thaliana* AT5G17310.2 UGP2 \| UDP-glucose pyrophosphorylase 2 | 73 | 0 | 709 |
| *AaTPS1* | 753 | *A. thaliana* AT1G78580.1 ATTPS1, TPS1 \| trehalose-6-phosphate synthase | 80 | 0 | 1018 |
| *AaTPS2* | 879 | *A. thaliana* AT1G68020.2 ATTPS6, TPS6 \| UDP-Glycosyltransferase / trehalose-phosphatase family protein | 69 | 0 | 1271 |
| *AaTPS3* | 860 | *A. thaliana* AT4G17770.1 ATTPS5, TPS5 \| trehalose phosphatase/synthase 5 | 69 | 0 | 1280 |
| *AaTPP1* | 365 | *A. thaliana* AT5G65140.1 TPPJ, TREHALOSE-6-PHOSPHATE PHOSPHATASE J | 65 | 1e^-107^ | 384 |
| *AaTPP2* | 411 | *A. thaliana* AT1G35910.1 ATTPPD, TPPD, TREHALOSE-6-PHOSPHATE PHOSPHATASE D | 55 | 1e^-105^ | 380 |
