# Supplementary material for: Carbohydrate-mediated responses during zygotic and early somatic embryogenesis in the endangered conifer, Araucaria angustifolia
Source: PLoS One. 2017 Jul 5;12(7):e0180051. doi: 10.1371/journal.pone.0180051 (PMC5497979; doi:10.1371/journal.pone.0180051)
Supplement: S5 Table — Values are presented in average ± standard deviation. (DOCX) [file pone.0180051.s008.docx]

**Table S5.** Relative gene expression of sugar sensing and trehalose biosynthesis pathway associated genes of zygotic embryo stages (GZE, CZE, MZE, CZEMG and MZEMG) and two embryogenic cultures in proliferation (SE1 and SE6) and maturation (S1M and S6M) phase of *A. angustifolia*. Values are presented in average ± standard deviation.

| *Samples** | *AaTOR* | *AaRAPTOR* | *AaLST8* | *AaSnRK1* | *AaUGP1* |
| --- | --- | --- | --- | --- | --- |
| *GZE* | 1.00 ± 0.08 A | 1.00 ± 0.07 A | 1.00 ± 0.05 A | 1.00 ± 0.09 B | 1.00 ± 0.08 D |
| *CZE* | 0.85 ± 0.10 AB | 0.75 ± 0.04 B | 0.75 ± 0.07 B | 0.21 ± 0.01 G | 0.90 ± 0.09 DE |
| *MZE* | 0.87 ± 0.07 AB | 0.74 ± 0.04 B | 0.59 ± 0.05 CD | 0.26 ± 0.04 FG | 1.07 ± 0.12 D |
| *CZEMG* | 0.67 ± 0.04 BC | 0.59 ± 0.07 BC | 0.74 ± 0.06 BC | 0.31 ± 0.02 EF | 0.53 ± 0.06 E |
| *MZEMG* | 0.73 ± 0.05 BC | 0.48 ± 0.04 CD | 0.93 ± 0.07 A | 0.24 ± 0.02 FG | 0.52 ± 0.05 E |
| *SE1* | 0.55 ± 0.04 CD | 0.42 ± 0.04 CD | 0.45 ± 0.02 DE | 0.39 ± 0.03 DE | 2.31 ± 0.07 B |
| *SE6* | 0.84 ± 0.11 AB | 0.94 ± 0.12 A | 0.47 ± 0.05 DE | 0.70 ± 0.04 C | 3.16 ± 0.39 A |
| *S1M* | 0.38 ± 0.04 D | 0.37 ± 0.02 D | 0.37 ± 0.04 E | 0.41 ± 0.06 D | 1.34 ± 0.12 CD |
| *S6M* | 1.02 ± 0.06 A | 0.59 ± 0.01 BC | 0.46 ± 0.04 DE | 1.56 ± 0.16 A | 1.73 ± 0.09 C |
|  |  |  |  |  |  |
| *Samples** | *AaTPS1* | *AaTPS2* | *AaTPS3* | *AaTPP1* | *AaTPP2* |
| *GZE* | 1.00 ± 0.12 A | 1.00 ± 0.04 A | 1.00 ± 0.06 A | 1.00 ± 0.12 F | 1.00 ± 0.17 BC |
| *CZE* | 0.43 ± 0.02 BC | 0.60 ± 0.03 C | 0.83 ± 0.05 B | 3.13 ± 0.20 D | 0.17 ± 0.01 E |
| *MZE* | 0.54 ± 0.03 B | 0.67 ± 0.11 C | 0.80 ± 0.09 B | 1.68 ± 0.10 E | 0.12 ± 0.01 F |
| *CZEMG* | 0.28 ± 0.04 D | 0.24 ± 0.01 EF | 0.59 ± 0.05 CD | 1.14 ± 0.07 F | 0.28 ± 0.01 D |
| *MZEMG* | 0.36 ± 0.03 CD | 0.25 ± 0.02 EF | 0.63 ± 0.07 C | 1.78 ± 0.29 E | 0.07 ± 0.00 G |
| *SE1* | 0.34 ± 0.03 CD | 0.43 ± 0.04 D | 0.48 ± 0.05 D | 7.47 ± 0.79 B | 0.88 ± 0.05 BC |
| *SE6* | 0.36 ± 0.02 CD | 0.83 ± 0.03 B | 0.51 ± 0.04 CD | 11.23 ± 2.04 A | 0.76 ± 0.07 C |
| *S1M* | 0.27 ± 0.02 D | 0.20 ± 0.02 F | 0.27 ± 0.02 E | 4.48 ± 0.21 C | 1.05 ± 0.04 B |
| *S6M* | 0.47 ± 0.05 BC | 0.35 ± 0.03 DE | 0.30 ± 0.02 E | 8.85 ± 0.75 AB | 2.05 ± 0.29 A |

^a^ Samples according to Figure
