# Supplementary material for: Carbohydrate-mediated responses during zygotic and early somatic embryogenesis in the endangered conifer, Araucaria angustifolia
Source: PLoS One. 2017 Jul 5;12(7):e0180051. doi: 10.1371/journal.pone.0180051 (PMC5497979; doi:10.1371/journal.pone.0180051)
Supplement: S1 Appendix — (DOCX) [file pone.0180051.s009.docx]

**S1 Appendix.** The protein sequences are conserved among the key players in sugar-mediated metabolic status

To identify putative genes associated with sugar sensing (*TOR, RAPTOR, LST8* and *SnRK1*) and the trehalose biosynthesis pathway (*UGP, TPS* and *TPP*) from *A. angustifolia,* a tBLASTn search was performed using *A. thaliana* protein sequences as queries against an *A. angustifolia* transcriptome database [1]. A single-copy of each gene was identified in *A. angustifolia* (genes designated as *AaTOR, AaRAPTOR, AaLST8, AaSnRK1* and *AaUGP1*), except for the *TPS* gene that had three copies (*AaTPS1, AaTPS2* and *AaTPS3*) and *TPP* that had two copies (*AaTPP1* and *AaTPP2*). A phylogenetic analysis was performed to identify the *A. angustifolia* homologs of these genes from Viridiplantae and Eukarya species to evaluate the diversity of sugar sensing and signaling proteins. Most of the trees obtained had a topology that was congruent with the established phylogenetic relationships of the constituent species [2] (S2A-G Fig).

The *AaTOR* gene is predicted to encode a protein of 1,856 amino acids with a molecular mass of 209.3 kDa. The phylogenetic study of AaTOR suggested that this protein is clustered with TOR from other plant species (S2A Fig) being 52% identical to AtTOR. Furthermore, this analysis showed that AaTOR is 33% identical to human TOR and 31.5 % and 31.8 % identical to yeast TOR (TOR1 and TOR2, respectively). The alignment of AaTOR with TOR protein sequences from other organisms revealed a high degree of conservation of the C-terminal region, which is composed of four main domains (FAT-FRB-kinase-FATC domains) (S3A Fig). We observed poorly conserved stretches of amino acids throughout the N-terminal region, more specifically in the HEAT repeat domain, which is found in all TOR proteins, except for in *A. angustifolia*, which have been proposed to be involved in protein interactions.

While *A. thaliana* has two copies of RAPTOR (*AtRAPTOR1 and AtRAPTOR2*), only one copy was found in *A. angustifolia*. *AaRAPTOR* encodes a protein of 1,375 amino acids with a predicted molecular mass of 150.68 kDa, and this protein has a Raptor N-terminal CASPase like domain well conserved beyond Eukaria and Viridiplantae (S3B Fig). The phylogenetic analysis indicated that *AaRAPTOR* is 64.8 % and 60.8 % identical to *AtRAPTOR1* and *AtRAPTOR2*, respectively, and 41.1 % identical to human *RAPTOR* (S2B Fig).

tBLASTn analysis using *A. thaliana* LST8 as the query identified a gene product of 1,966 bp in the Araucaria Transcriptome database [1]. The *AaLST8* gene encodes a protein of 316 amino acids with a predicted molecular mass of 35.57 kDa. The phylogenetic analysis indicated that AaLST8 is clustered with LST8 from other plant species (S2C Fig), being 81.3 and 65.3% identical to AtLST8.1 and AtLST8.2, respectively, and 45.7 % identical to human LST8. The AaLST8 protein is composed of six tryptophan-aspartic (WD) repeat signatures (S3C Fig) and, based on a modeling study of the protein, the predicted structure of AaLST8 is very similar to the beta-propeller fold of WD-repeat proteins.

As well as AaTOR, AaRAPTOR and AaLST8, the phylogenic tree for AaSnRK1 was congruent with the established phylogenetic relationships of the constituent species, being 76.8 %, 74.3 % and 62.6 % identical to *A. thaliana* SnRK1.1, SnRK1.2 and SnRK1.3, respectively (S2D Fig). The *AaSnRK1* gene encodes a protein of 511 amino acids with a predicted molecular mass of 58.42 kDa. Alignment of AaSnRK1 with plant SnRK1, human AMPK and yeast Snf1 protein sequences showed a high degree of conservation of the N-terminal region, which is composed of the STKc AMPK alpha domain (S3D Fig).

Two putative UGP genes were found in *A. angustifolia*. *AaUGP1* and *AaUGP2* encode proteins with predicted molecular masses of 52.93 kDa and 100.85 kDa, respectively. However, the phylogenetic analysis suggested that there is a functional divergence between AaUGP1 and AaUGP2, since they grouped in different clusters in the tree topology (S2E Fig). Furthermore, AaUGP1 has a Viridiplantae conserved UGPase domain, and is 75.4 % and 76.7 % identical to AtUGP1 and AtUGP2, respectively. AaUGP2 has an amino acid sequence identity of 50.2 % with AtUGP3 and UDP-N-acetylglucosamine (UDPGlcNAc) pyrophosphorylase domain, which is fundamental during N- and O-linked glycosylations [3]. Accordingly, *AaUGP2* was not used in the subsequent analysis.

Since plant TPS and TPP proteins are encoded by multi-gene families, building a phylogenetic tree using sequences from several members of the Viridiplantae is computationally challenging. Thus, based on previous studies of these proteins, new phylogenetic trees were generated containing *A. angustifolia* and *P. pinaster* homologs. [4] showed that the TPS family can be divided into class I and class II according to the structural characteristics of the gene (size of introns and active domain of the protein). [4] used accessions of *A. thaliana, Populus trichocarpa* and *Oryza sativa*, and our tree topology was in accordance with that described by these authors, with one *A. angustifolia* gene classified as class I (*AaTPS1*) and two as class II (*AaTPS2* and *AaTPS3*) (S2F Fig). The same strategy was used to construct the TPP phylogenetic tree, using the initial parameter construction reported by [5], who used the same species as those in [4]. Again, the tree topology was conserved between that shown by [5], and the tree made using the putative *A. angustifolia* (*AaTPP1* and *AaTPP2*) genes (S2G Fig).

All sequences used in this study were obtained from a *de novo* transcriptome database [1]. These analyses improve the knowledge of protein functions during embryogenetic development, related to genes involve in sugar-mediated metabolic status. The results also enhance the knowledge of sugar sensing and signaling during embryogenesis of a basal gymnosperm with recalcitrant seeds [6].

**References**

1. Elbl P, Campos RA, Lira BS, Andrade SCS, Jo L, dos Santos ALW, et al. Comparative transcriptome analysis of early somatic embryo formation and seed development in Brazilian pine, *Araucaria angustifolia* (Bertol.) Kuntze. Plant Cell Tiss Org. 2015a 120: 903–915.
2. Stevens PF. Angiosperm Phylogeny Website. 2001; Version 12, July 2012.
3. Okazaki Y, Shimojima M, Sawada Y, Toyooka K, Narisawa T, Mochida K, et al. 2009. A chloroplastic UDP-glucose pyrophosphorylase from Arabidopsis is the committed enzyme for the first step of sulfolipid biosynthesis. The Plant Cell. 2009; 21: 892-909.
4. Yang HL, Liu YJ, Wang CL, Zeng QY. Molecular evolution of trehalose-6-phosphate synthase (TPS) gene family in Populus, Arabidopsis and rice. PloS One. 2012; 7: e42438. doi: 10.1371/journal.pone.0042438.
5. Vandesteene L, López-Galvis L, Vanneste K, Feil R, Maere S, Lammens W, et al. Expansive evolution of the trehalose-6-phosphate phosphatase gene family in Arabidopsis. Plant Physiol. 2012; 160: 884-896.
6. dos Santos ALW, Elbl P, Navarro BV, de Oliveira LF, Salvato F, Balbuena TS, *et al*. Quantitative proteomic analysis of *Araucaria angustifolia* (Bertol.) Kuntze cell lines with contrasting embryogenic potential. J. Proteomics. 2016; 130: 180-189.
